# Moisture-Resistant Scalable Ambient-Air Crystallization of Perovskite Films via Self-Buffered Molecular Migration Strategy

**DOI:** 10.1007/s40820-025-01851-9

**Published:** 2025-09-01

**Authors:** Mei Yang, Weidong Zhu, Laijun Liang, Wenming Chai, Xiaomeng Wu, Zeyang Ren, Long Zhou, Dazheng Chen, He Xi, Chunfu Zhang, Jincheng Zhang, Yue Hao

**Affiliations:** 1 State Key Laboratory of Wide-Bandgap Semiconductor Devices and Integrated Technology, Xidian University, 710071 Xi’an, People’s Republic of China; 2 School of Electronic Engineering, Xi’an Shiyou University, 710065 Xi’an, People’s Republic of China; 3 School of Advanced Materials and Nanotechnology, Xidian University, 710126 Xi’an, People’s Republic of China

**Keywords:** Perovskite solar cell, Ambient-air annealing, Intermediate phase, Intermolecular exchange, High-humidity crystallization

## Abstract

Ambient-air, moisture-assisted annealing is widely used in fabricating perovskite solar cells (PSCs). However, the inherent sensitivity of perovskite intermediate-phase to moisture—due to fast and spontaneous intermolecular exchange reaction—requires strict control of ambient humidity and immediate thermal annealing treatment, raising manufacturing costs and causing fast nucleation of perovskite films. We report herein a self-buffered molecular migration strategy to slow down the intermolecular exchange reaction by introducing a n–butylammonium bromide shielding layer, which limits moisture diffusion into intermediate-phase film. This further endows the notably wide nucleation time and humidity windows for perovskite crystallization in ambient air. Consequently, the optimized 1.68 eV-bandgap n-i-p structured PSC reaches a record-high reverse-scan (RS) PCE of 22.09%. Furthermore, the versatility and applicability of as-proposed self-buffered molecular migration strategy are certified by employing various shielding materials and 1.53 eV-/1.77 eV-bandgap perovskite materials. The n-i-p structured PSCs based on 1.53 eV- and 1.77 eV-bandgap perovskite films achieve outstanding RS PCEs of 25.23% and 19.09%, respectively, both of which are beyond of the state-of-the-art ambient-air processed PSCs.

**Supplementary Information:**

The online version contains supplementary material available at 10.1007/s40820-025-01851-9.

## Highlights

- A self-buffered molecular migration strategy is developed to suppress spontaneous intermolecular exchange between perovskite intermediate phase and ambient moisture.
- Exceptionally broad nucleation time and humidity tolerance windows are achieved for perovskite crystallization under ambient air conditions. 1.68 eV-bandgap perovskite solar cells (PSCs) reach a record efficiency of 22.09% when processed in 50–60% relative humidity.
- The strategy is broadly applicable to 1.53 eV- and 1.77 eV-bandgap perovskite films, enabling high-efficiency PSCs via air-based crystallization processing.

**Supplementary Information:**

The online version contains supplementary material available at 10.1007/s40820-025-01851-9.

## Graphical abstract

**Figure:**
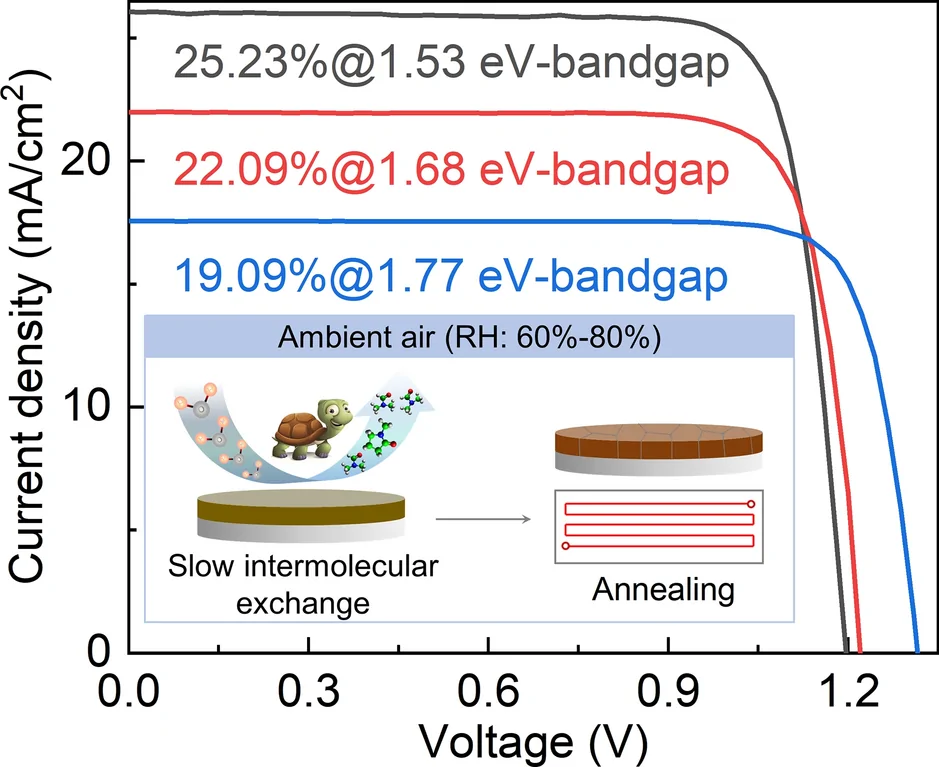

## Introduction

Significant advancements in the power conversion efficiency (PCE) and stability of perovskite solar cells (PSCs) have garnered substantial research interest in the field of photovoltaic technologies [1].The PCE has risen from 3.81% in 2009 to a certified value of 26.7% in 2024 [1, 2]. In addition, the low-cost and abundant raw materials, relatively modest purity requirements, and simple solution-processing methods make PSCs a promising alternative to silicon-based solar cells in terms of cost-effectiveness [3, 4]. It is widely acknowledged that achieving high-performance PSCs requires the growth of high-quality, stable, and uniform perovskite films [2, 5, 6]. Numerous studies have shown that both the growth dynamics and stability of perovskite films are highly sensitive to environmental factors, particularly moisture [7–9]. Moisture can disrupt the organic–inorganic interactions within the perovskite materials, leading to their degradation into PbI_2_ [10, 11]. In this case, the preparation of perovskite films requires precise control of the surrounding atmosphere, and hence, many high-efficiency devices can only be fabricated in nitrogen-filled gloveboxes, which, unfortunately, increases production costs [12–14].

With increasing efforts to develop high-performance PSCs in ambient air, it has been found that the controlled moisture levels can improve film morphology and crystallinity, but these effects are largely valid during the ambient-air annealing stage [15–18]. For example, Yang et al. [19] reported for the first time a growth mode via thermal annealing of the perovskite intermediate-phase film in a humid environment (e.g., ambient air) to greatly improve the film quality, grain size, carrier mobility, and lifetime. And, they proposed that when annealed in the air, highly hygroscopic methylammonium (MA) cations pull moisture from the environment and then lead to the partial dissolution of the perovskite grains, finally enlarging their sizes [20]. Huang et al. [21] used in situ grazing-incident wide-angle X-ray scattering (GIWAXS) to capture the phase transformation of perovskite films during annealing under different relative humidity (RH) conditions, from which they reckoned that a moderate water content accelerates the crystal formation and enhances the texture orientation of the films. Liu et al. [8] designed air exposure-free characterization techniques to demonstrate that a controllable moisture treatment for perovskite intermediate-phase can promote the mass transportation of organic salts, and translates to high-quality perovskites with much-suppressed defects. Zhong et al. [9] showed that ambient moisture can induce the secondary crystal growth to simultaneously improve the crystalline quality and optimize the energy level of perovskite film. Lee et al. [22] showed that the presence of atmospheric humidity is perhaps indispensable during processing, and fundamentally underlies the reproducibility of phase-stable and high-performance CH(NH_2_)_2_PbI_3_ (FAPbI_3_) PSCs. Collectively, these reports make clear that the proper moisture-assisted thermal annealing in ambient air indeed promotes the growth of perovskite films with fewer defects and larger grains.

Ambient-air, moisture-assisted thermal annealing typically involves transferring a perovskite intermediate-phase film, prepared by spin-coating, blade-coating, or slit-coating method, from an anhydrous environment (< 1 ppm) to a preheated hotplate (≥ 100 °C) in ambient air, followed by immediate thermal annealing for 10–60 min. Perovskite intermediate-phase films are highly sensitive to atmospheric moisture [23, 24], as exposure to ambient air triggers rapid and spontaneous intermolecular exchange between residual solvent molecules and moisture [25–28]. This chemical reaction can lead to the fast crystallization of perovskite grains, but it also hinders the formation of high-quality films [29–31]. For instance, Wei et al. [8] showed that prolonged exposure of intermediate-phase films to ambient air without thermal annealing resulted in the interaction of water molecules with the surface grains, forming metastable hydrated intermediate phases that are highly reactive and may transform into *δ*-phase perovskite or decompose into PbI_2_. Hence, in order to utilize the beneficial effects of ambient-air annealing, the RH must be controlled within 30–40%, and films should be thermally annealed immediately after exposure [29, 32, 33]. These exacting humidity and time requirements not only bring additional constraints for environment and recipes controls, but also significantly shorten down the nucleation stage of perovskite films, with risks of the formation of impurity phases and pinholes, the confined grain coarsening, etc. Additionally, seasonal humidity fluctuations can further increase manufacturing costs [16]. Recently, Gao et al. [16] reported the successful modulation of ambient-air crystallization behavior in FA_0.95-x_MA_x_Cs_0.05_PbI_3_ films by spin-coating a water ethyl acetate solution prior to annealing. Zhang et al. [14] utilized bio-derived chitin-based polymers to stabilize the lead iodide and organic salt precursor inks, achieving the highly crystalline and oriented perovskite films with fewer detrimental charge defects under open-air conditions. Yu et al. [34] introduced a two-dimensional material, MBene, into a green antisolvent to simultaneously regulate crystallization and passivate defects in perovskites, enabling the fabrication of PSCs with an efficiency of 24.22% under ambient air with a RH of 50%–60%. Wang et al. [35] demonstrated that incorporating sodium benzene phosphinate as an additive enables the blade-coating fabrication of high-performance, stable wide-bandgap PSCs in ambient air with an RH of ~ 60%. Despite these advancements, careful control of the ambient exposure time prior to annealing remains essential. Moreover, the humidity window for achieving high-performance and reproducible PSCs under ambient conditions is still narrow. Therefore, it is crucial to develop strategies that reduce the sensitivity of perovskite intermediate-phase films to moisture, enabling versatile nucleation control under varying ambient humidity conditions.

In this study, we propose a self-buffered molecular migration strategy to slow down the spontaneous intermolecular exchange between one-step processed perovskite intermediate-phase films and ambient humidity by spin-coating n–butylammonium bromide (BABr) molecules on their surface before ambient-air annealing. The BABr coating acts as a barrier, preventing the diffusion of ambient moisture into the intermediate-phase film, thereby facilitating a more relaxed intermolecular exchange. This significantly broadens the nucleation time and humidity windows for perovskite crystallization in ambient air. As a result, the 1.68 eV-bandgap perovskite films with a pure phase, fewer defects, larger-sized grains, and better stability are obtained relaxedly in ambient air with a high RH of 60%–80%. Under the optimized ambient-air exposure time of 30 min and RH of 50%–60%, the wide-bandgap n-i-p structured PSCs reach an enhanced average reverse-scan (RS) PCEs of (21.72 ± 0.24)% and superior humidity stability, wherein the best-performing one yields an impressive value of 22.09% (RS), setting a new record for PCE in ambient-air processed 1.68 eV-bandgap devices. Furthermore, we demonstrate that other materials, such as methylammonium chloride (MACl), 4-trifluoromethyl–phenylammonium chloride (CF_3_–PEACl), phenylethylammonium chloride (PEACl), trifluoromethyl 2-phenylethylamine hydroiodide (CF_3_–PEABr), and octylammonium bromide (OABr), can induce similar beneficial effects as BABr. This strategy is also applicable to other typical perovskite films. Using this approach, we fabricate n-i-p structured PSCs with outstanding RS PCEs of 25.23% and 19.09% based on 1.53 eV- and 1.77 eV-bandgap perovskite films, both of which rank among the highest efficiency PSCs reported to date.

## Experimental Section

### Materials and Reagents

Unless otherwise stated, all materials were used as received, without further purification. CsI (ultra-dry, 99.998% (metal basis)), PbBr_2_ (ultra-dry, 99.999% (metal basis)), PbCl_2_ (ultra-dry, 99.999% (metal basis)), PbI_2_ (ultra-dry, 99.999% (metal basis)), and a SnO_2_ aqueous solution (15% in H_2_O colloidal dispersion liquid) were purchased from Alfa-Aesar. N, N-Dimethylformamide (DMF, anhydrous, 99.8%), dimethyl sulfoxide (DMSO, anhydrous, 99.9%), isopropanol (IPA, ACS reagent, ≥ 99.5%), bis (trifluoromethane) sulfonimide lithium salt (Li–TFSI, 99.95%, trace metals basis), and chlorobenzene (CB, anhydrous, 99.8%) were purchased from Sigma-Aldrich. Tris(2-(1H-pyrazol-1-yl)-4-tert–butylpyridine)-cobalt(III) tris (bis (trifluoromethylsulfonyl) imide) (FK209, Co(III) TFSI), methylammonium bromide (MABr, > 99.99%), and formamidinium iodide (FAI, > 99.99%) were purchased from Greatcell Solar Materials Pty Ltd. 1-Methyl-2-pyrrolidinone (NMP 99%) and acetonitrile (99.9%) were purchased from J&K Scientific. Pb(SCN)_2_ (99.5%), N-butylammonium bromide (BABr), 4-tert–butylpyridine (TBP, 96%), 2,2’,7,7’-tetrakis (N,N-di-p-methoxyphenylamine)-9,9 spirobifluorene (Spiro–OMeTAD, 99.8%), methylammonium chloride (MACl, 99.5%) phenylethylammonium chloride (PEACl, 99.5%), octylammonium bromide (OABr, 99.5%), 4-trifluoromethyl–phenylammonium chloride (CF_3_–PEACl, 99.5%), and trifluoromethyl 2-phenylethylamine hydroiodide (CF_3_–PEABr, 99.5%) were purchased from Xi’an Yuri Solar (Xi’an, China). Patterned ITO glass (2 × 2.5 cm^2^ in size) was purchased from Yingkou OPV Tech New Energy Co. Ltd.

### Preparation of Precursors

1.68 eV-bandgap FA_0.65_MA_0.20_Cs_0.15_PbI_2.4_Br_0.6_ precursor solution was prepared by adding 479.45 mg of PbI_2_, 145.30 mg of FAI, 95.42 mg of PbBr_2_, 29.11 mg of MABr, and 50.66 mg of CsI in 1 mL mixed solvent of DMF/NMP (4:1 by volume). 1.53 eV-bandgap perovskite Cs_0.025_MA_0.075_FA_0.90_PbI_3_ precursor solution was prepared by dissolving 20.784 mg of CsI, 247.68 mg of FAI, 12.72 mg of MAI, 15 mg of MACl, 11.124 mg of PbCl_2_, and 785.55 mg of PbI_2_ in 1 mL mixed solvent of DMF/DMSO (4:1 by volume). 1.77 eV-bandgap perovskite FA_0.8_Cs_0.2_Pb(I_0.6_Br_0.4_)_3_ precursor solution was cooked by dissolving 62.35 mg of CsI, 165.09 mg of FAI, 264.25 mg of PbBr_2_, and 221.28 mg of PbI_2_ in 1 mL mixed solvent of DMF/DMSO (4:1 by volume). All the resultant precursors were added with 10 mg mL^−1^ of Pb(SCN)_2_ and filtered through a 0.45 μm PTFE filter before use. BABr solution was obtained by adding 2 mg mL^−1^ BABr into IPA. MACl, CF_3_–PEACl, PEACl, CF_3_–PEABr, or OABr solution was prepared by dissolving 1.5 mg MACl, CF_3_–PEACl, PEACl, CF_3_–PEABr, or OABr into IPA, respectively. SnO_2_ nanocrystal solution was obtained by diluting the commercial SnO_2_ nanocrystal colloids (15% in H_2_O colloidal dispersion) with deionized water (1:4 by volume). Spiro–OMeTAD solution was prepared by dissolving 72.3 mg of Spiro–OMeTAD in 1 mL of CB with 16.3 µL of Li–TFSI/acetonitrile solution (520 mg mL^−1^), 28.5 µL of TBP, and 15.1 µL of FK209 Co(III) TFSI/acetonitrile solution (375 mg mL^−1^).

### Fabrication of PSCs

ITO glass substrate was ultrasonically cleaned in Decon90, deionized water, acetone, and anhydrous ethanol for 20 min, followed by nitrogen blowing and ultraviolet ozone treatment for 30 min. SnO_2_ aqueous solution was spin-coated onto the cleaned ITO substrate at 3000 r min^−1^ for 30 s and annealed in ambient-air atmosphere at 150 °C for 30 min to deposit the SnO_2_ electron transporting layer (ETL). No further treatment is needed for the SnO_2_ ETL, and it was directly transferred into a nitrogen-filled glovebox to prepare perovskite intermediate film. Typically, 75 μL of perovskite Cs_0.15_MA_0.20_FA_0.65_(Br_0.20_I_0.80_)_3_ precursor solution was spin-coated onto the SnO_2_ ETL at 1000 r min^−1^ for 5 s and at 4000 r min^−1^ for 45 s. At the 12-s mark, 200 μL of CB, acting as an antisolvent to DMF and NMP solvent, was dropped onto the sample. After the above spin-coating process, 50 μL of BABr solution was further spin-coated onto the sample at 1000 r min^−1^ for 5 s and 4000 r min^−1^ for 25 s. After that, the sample was transferred to ambient air with controlled RH of 60%–80% and stood for 0 or 15 min before thermal annealing at 100 °C for 10 min in the same ambient air to obtain the crystallized Cs_0.15_MA_0.20_FA_0.65_(Br_0.20_I_0.80_)_3_ film. Then, 80 μL of spiro–OMeTAD solution was spin-coated onto as-obtained Cs_0.15_MA_0.20_FA_0.65_(Br_0.20_I_0.80_)_3_ film at 1000 r min^−1^ for 5 s and 4000 r min^−1^ for 45 s to form the hole transporting layer (HTL). Finally, the 100 nm silver layer with the specific area of 0.1 cm^2^ was thermally evaporated to form the metal electrode. Thus, the target 1.68 eV-bandgap PSC was obtained. The control 1.68 eV-bandgap PSCs were fabricated according to the recipes like the above, wherein the only difference is that they were free of spin-coating the BABr solution. The basic preparation recipes for the target 1.53 eV-bandgap and 1.77 eV-bandgap perovskite films as well as the PSCs are like the target 1.68 eV-bandgap ones. The as-obtained PSCs were routinely stored in a drying cabinet with RH below 10%, unless otherwise specified.

### Characterizations

The sample weight variation in ambient air was monitored in situ by a precision electronic analytical balance (Sartorius, Secura 225D-1CN, 0.01 mg). Fourier transform infrared spectroscopy (FTIR) spectra were measured using a Bruker-VERTEX 70 instrument. Scanning electron microscope (SEM) images were obtained using a desktop scanning electron microscope (Phenom Pro, Thermo Fisher Scientific). X-ray diffraction (XRD) tests were performed using an X’Pert^3^ powder X-ray diffractometer (Malvern PANalytical, Malvern, UK). Absorption spectra were recorded by an ultraviolet–visible (UV–Vis) spectrophotometer (U-4100, Hitachi, Tokyo, Japan). Steady-state photoluminescence (PL) and time-resolved photoluminescence (TRPL) were measured using a Fluo Time 300 spectrometer (PicoQuant, Berlin, Germany). X-ray photoelectron spectroscopy (XPS) and ultraviolet photoelectron spectroscopy (UPS) tests were fulfilled by X-ray photoelectron spectroscopy (Nexsa, Thermo Fisher Scientific, Waltham, MA, USA). Contact angle images were obtained using a contact angle-measuring instrument (SCI3000F). GIWAXS measurements were carried out using a Xeuss 2.0 SAXS/WAXS laboratory beamline with a copper X-ray source (8.05 keV, 1.54 Å). In situ fluorescence scattering absorption spectra and PL processes were monitored by a Du-100 dynamic spectrometer system. Current density versus voltage (J-V) curves were recorded using a Keithley 2450 source meter unit (Tektronix, Beaverton, OR, USA) under AM 1.5G illumination (100 mW cm^−2^) simulated by the XES-70 S1 solar simulator. For the maximum power point tracking (MPPT) tests, the switch of the device from dark condition to light illumination was achieved by directly turning on the power switch of solar simulator. A shadow mask with an area of 0.09 cm^2^ was adopted to confine the active area of the PSCs during the tests. Electrochemical impedance spectroscopy (EIS) and Mott–Schottky (M-S) tests were performed on an electrochemical workstation (CHI660B, CH Instruments, Austin, TX, USA) in the dark, and a 1 V forward bias was applied for AC impedance measurement. Transient photocurrent (TPC) study was accomplished by a home-built system. The sample was excited by a 532 nm pulse laser (1000 Hz, 3.2 ns). Transient photovoltage (TPV) tests were carried out on the same system, while the sample was excited by a 405 nm pulse laser (50 Hz, 20 ms). External quantum efficiency (EQE) spectra were acquired using a monochromator (Cornerstone 74,004, Newport) equipped with a 150 W xenon lamp (Oriel). Apart from the long-term stability test, the other device performance tests and characterizations were performed in ambient air free of specific RH controlling.

## Results and Discussion

### Illustration of Self-Buffered Molecular Migration and Its Effects on Perovskite Intermediate-Phases

**Fig. 1:**
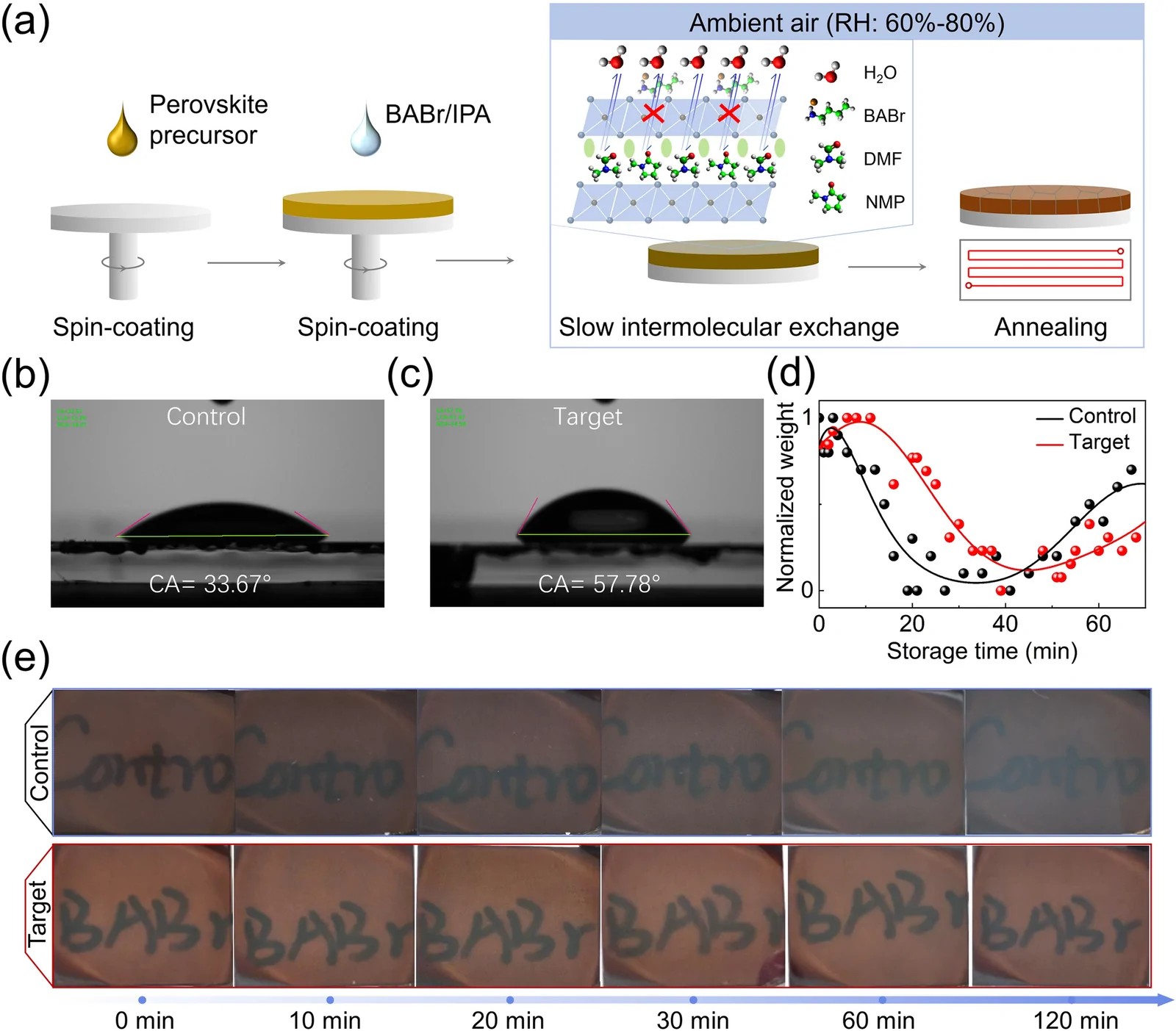
**a** Schematic of the experimental procedures involved in the self-buffered molecular migration strategy. Water contact angles of the **b** control and **c** target perovskite intermediate-phase films before annealing. **d** Weight changes of the control and target perovskite intermediate-phase films during ambient-air exposure. **e** Photographs of the control and target intermediate-phase films exposed to ambient air for varying time periods (0 to 120 min)

The basic experimental procedures involved in the self-buffered molecular migration strategy are illustrated briefly in Fig. 1a and described in detail in the experimental section. Initially, a perovskite intermediate-phase film was produced using an antisolvent assisted one-step spin-coating method in a N_2_-filled glovebox. Next, the BABr solution with a typical concentration of 2 mg mL^−1^ was spin-coated onto the intermediate-phase film to form a surface shielding layer. This sample is referred to as the target intermediate-phase film, while the one without the BABr coating is designated as the control. The BABr coating increases the water contact angle from 33.67 to 57.78° (Fig. 1b, c). Subsequently, the sample was removed from the glovebox and exposed to ambient air at room temperature for varying durations. During this static settling period, intermolecular exchange between the perovskite intermediate-phase film and ambient humidity occurred spontaneously [26, 36–38], as evidenced by a clear weight loss of the film Fig. 1d. In contrast, the BABr coating effectively suppressed this weight loss, indicating a slowed intermolecular exchange process. The relatively weaker color changes observed over time in the film with BABr coating can also support this fact Fig. 1e. Moreover, FTIR analysis showed that residual DMF/NMP solvent molecules were present exclusively in the target intermediate-phase film after 120 min of ambient-air exposure. (Fig. S1), further confirming the decelerated intermolecular exchange between residual solvent molecules and moisture for the target sample. Finally, the sample was annealed at 100 °C for 10 min in the similar ambient-air atmosphere to obtain the target perovskite film. A comparable perovskite film without the BABr coating was prepared as a control sample. To demonstrate the feasibility of the self-buffered molecular migration strategy, the ambient-air exposure and thermal annealing procedures were fulfilled in ambient air with a high RH of 60–80%, unless otherwise specified.

To further investigate the decelerated intermolecular exchange between the perovskite intermediate-phase film and ambient humidity induced by the BABr coating, we measured the XRD patterns of both the control and target intermediate-phase films, initially exposed to ambient air and after being exposed for approximately 10 min. As shown in Fig. S2a, distinct XRD peaks at 2*θ* = 10.56°, 13.11°, 26.20°, and 30.45° were observed in both samples, which can be attributed to the intermediate-phase species [37–40]. For the film exposed to ambient air, the control sample exhibited much stronger XRD peaks, which became even more pronounced after 10 min of exposure. In contrast, the target sample showed much weaker XRD peaks immediately after exposure to ambient air, and these peaks decreased slightly after 10 min of exposure. These XRD results clearly support the idea that the BABr coating retards the spontaneous intermolecular exchange between residual solvent molecules and ambient moisture in the target intermediate-phase film [24, 38]. Additionally, as shown in Fig. S2b, no 2D perovskite species were detected in either of the target films, since BABr-based 2D perovskites typically produce XRD peaks at 2*θ* < 10° [41–43]. This suggests that the BABr coating likely remains on top of the intermediate-phase film in its original form before thermal annealing, rather than reacting with the intermediate phase to form 2D perovskites. We therefore attribute the retardation of intermolecular exchange to the chemically inert and hydrophobic –NH_3_⁺ group in the BABr molecules, which prevents moisture from diffusing into the intermediate-phase film. Furthermore, the BABr coating acts as a barrier that limits the escape of residual solvent molecules from the intermediate-phase film, further suppressing the intermolecular exchange process.

### Promoted Film Quality and Stability by Self-buffered Molecular Migration

**Fig. 2:**
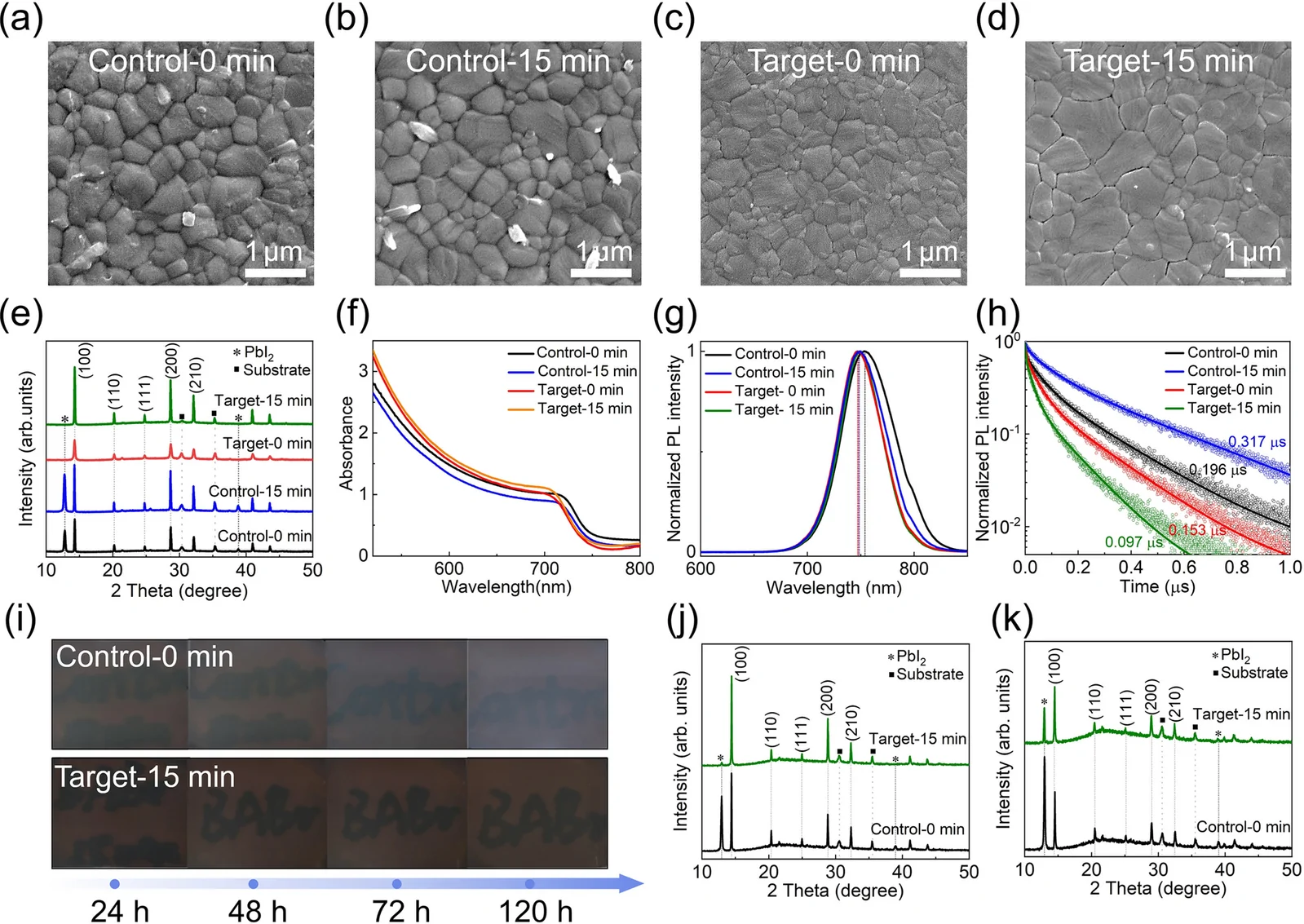
**a-d** SEM images, **e** XRD patterns, **f** UV–vis absorption spectra, **g** normalized steady-state PL spectra, and **h** TRPL results for control and target perovskite films obtained with 0- and 15-min ambient-air exposure. The TRPL spectra in panel **h** were measured from the samples grown on ITO/SnO_2_ substrates. **i** Photographs of the control and target perovskite films exposed to ambient air with RH of 60–80% for 24 to 120 h. XRD patterns of control and target perovskite films **j** after the 120-h ambient-air exposure and **k** after continuous simulated AM 1.5G illumination and 85 °C vapor fumigation for 60 min

To investigate the effects of slow intermediate-phase intermolecular exchange induced by self-buffered molecular migration strategy, the typical 1.68 eV-bandgap perovskite FA_0.65_MA_0.2_Cs_0.15_PbI_2.4_Br_0.6_ material was chosen, given its promising potential for silicon/perovskite tandem solar cells [44]. SEM images in Fig. 2a, b manifest that the ambient-air residence before annealing has a margin impact on the morphology of the control films. The average grain sizes for the control samples with 0- and 15-min ambient-air exposure are approximately 496 and 477 nm, respectively (Fig. S3). Impurity phases with bright contrast can be seen around the grain boundaries of both control samples, which are attributed to PbI_2_ species, as previously reported [44, 45]. Furthermore, the PbI_2_ phase becomes more prominent in the control sample with 15 min of ambient-air exposure. These observations are supported by the XRD patterns in Fig. 2e, where a clear XRD peak at 2*θ* = 12.79°, corresponding to PbI_2_, is evident in the control sample with 0 min of ambient-air exposure, and the peak intensity increases significantly for the sample with 15 min of exposure. For the control samples, rapid intermolecular exchange occurs between the intermediate-phase film and ambient humidity upon removal from the glovebox. This energetic exchange leads to minimal morphological changes in the final perovskite films but promotes the formation of the PbI_2_ impurity phase [36, 37]. Notably, the PbI_2_ impurity phase almost disappears when similar control samples are prepared in ambient air with a RH below 20% (Fig. S4).

In contrast, the slow intermediate-phase intermolecular exchange induced by self-buffered molecular migration strategy significantly influences the properties of the resulting perovskite films. The target film with 0 min of ambient-air exposure exhibits grain sizes comparable to those of the control sample, but it is free from the PbI₂ impurity phase (Fig. 2c), as further confirmed by the corresponding XRD pattern in Fig. 2e. The SEM and XRD results for a sample prepared in an N₂-filled glovebox for comparison are shown in Fig. S5a–c. The target film exposed in ambient air for 0 min shows nearly identical grain size and crystallinity to the film produced in the glovebox, despite being annealed in ambient air. Moreover, an additional reference sample was prepared by spin-coating pure IPA solvent. When this sample was removed from the glovebox and immediately annealed (exposed in ambient air for 0 min), it exhibited similar morphological and crystalline characteristics to the control sample with 0-min ambient-air exposure (Fig. S6), indicating that the IPA solvent had a negligible effect on the film properties. These findings collectively confirm the effectiveness of the BABr layer in shielding the intermediate-phase perovskite film from ambient humidity, thereby preventing intermolecular exchange with the surrounding humidity. When the ambient-air exposure time is extended to 15 min, larger grains are observed in the resulting film (Fig. 2d), with an average grain size of approximately 750 nm (Fig. S7). Moreover, its (100) and (200) XRD peaks at 2*θ* = 14.28° and 28.64° become significantly stronger than the target film with 0-min ambient-air exposure, indicating its higher crystallinity with fewer intragranular and grain boundaries [44, 46]. Additionally, the (100) and (200) XRD peaks at 2*θ* = 14.28° and 28.64°, respectively, are significantly more intense than those of the target film with 0 min of ambient-air exposure, indicating a higher degree of crystallinity with fewer intragranular defects and grain boundaries.

The basic optoelectronic properties of the control and target films prepared with 0- and 15-min ambient-air exposure were further investigated. The UV–vis absorption spectra, presented in Fig. 2f, reveal that both the control and target samples—regardless of 0- or 15-min ambient-air exposure—display a similar absorption onset wavelength at ~ 738 nm and a bandgap of approximately 1.68 eV (Fig. S8) [31, 44, 46], wherein the slight differences between them can be attributed to the introduced BABr molecules, with the additional Br⁻ anions incorporated into the target films. For the control samples, the one produced with 15-min ambient-air exposure exhibits lower absorption intensities compared to the one with 0-min exposure. This reduction is primarily attributed to the increased presence of PbI_2_ species. In contrast, the absorption intensities of the target samples increase, likely due to the complete elimination of PbI_2_ impurities and the enhanced crystallinity. To evaluate the carrier dynamics of the perovskite films grown on SnO_2_/ITO substrates, steady-state PL and TRPL decay tests were conducted. As shown in Fig. 2g, the steady-state PL peaks for both target films, prepared with 0 and 15 min of ambient-air exposure, shift to 747 nm. This shift contrasts with the control samples, which have PL peaks at 754 and 749 nm, respectively. The blue-shift in PL for the target samples is primarily attributed to a reduction in shallow-level defects associated with structural or crystalline imperfections [47]. Meanwhile, the TRPL results in Fig. 2h show average decay times of 0.196 and 0.317 μs for the control samples with 0- and 15-min exposure, respectively, while the target samples exhibit faster decay times of 0.153 and 0.097 μs. Faster TRPL decay typically indicates more efficient carrier transfer from the perovskite to the SnO_2_ layer [44, 47]. Hence, one can make sense that the target samples, particularly the one with 15-min ambient-air exposure, exhibit superior interfacial carrier transport characteristics, which could contribute to reduced non-radiative recombination of photo-generated carriers near the perovskite/SnO_2_ interface. In addition, the stability features of the control sample (0 min of ambient-air exposure) and the target sample (15 min of exposure) were also examined. As seen from Fig. 2i, after 120 h of exposure in ambient air with RH between 60% and 80%, the surface of the control sample undergoes whitening, while the target sample shows minimal color change. XRD patterns in Fig. 2j reveal that more PbI_2_ species are generated in the control sample after the 120-h exposure, indicative of perovskite decomposition [10, 11]. In a combined humidity, light, and thermal stress test involving continuous simulated AM 1.5G illumination and 85 °C vapor fumigation for 60 min, the XRD patterns in Fig. 2k show that the decomposition of the target sample is considerably weaker than that of the control sample. These results highlight the enhanced stability of the target perovskite film against humidity, light, and thermal stresses.

**Fig. 3:**
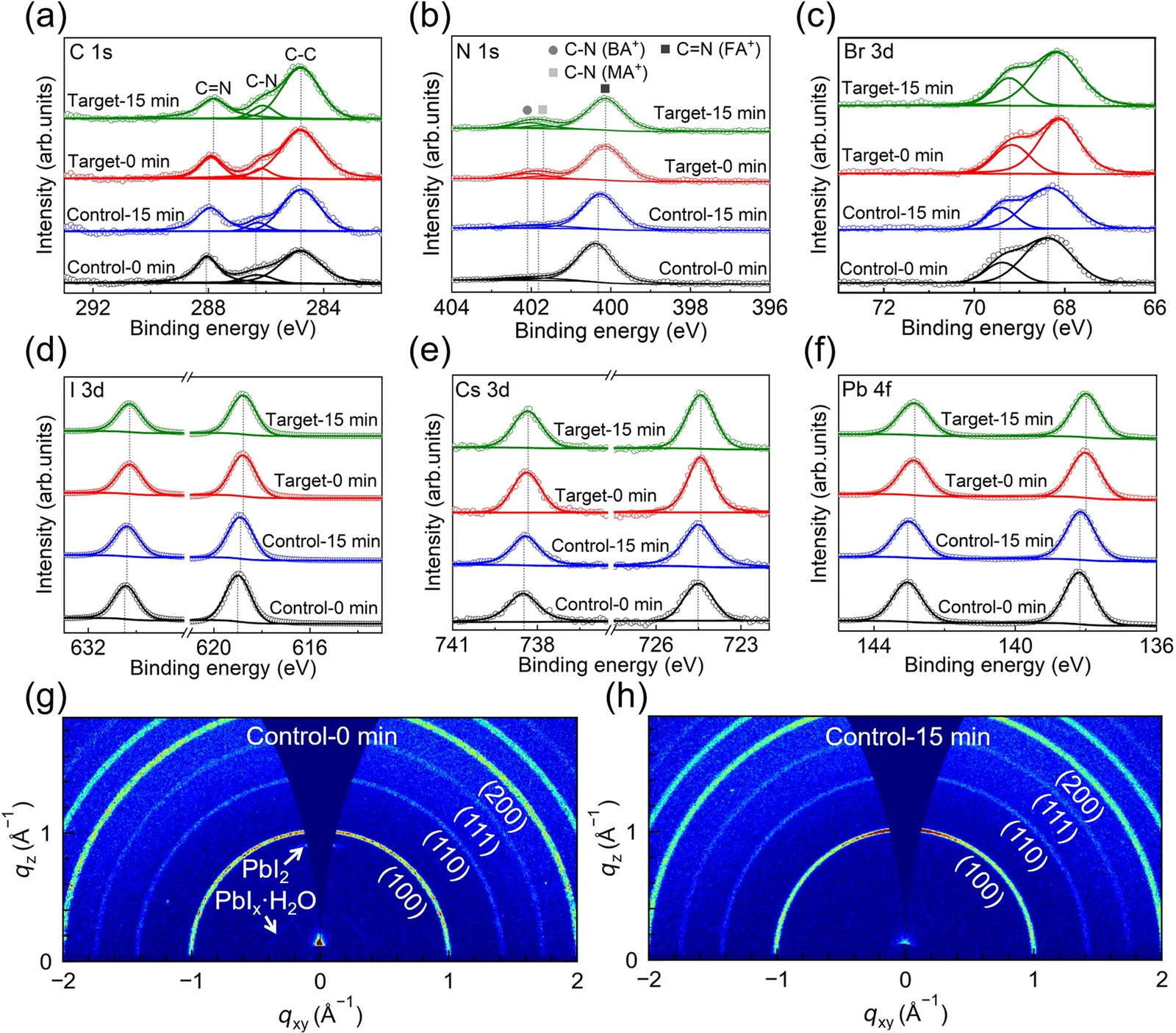
**a** C 1*s*, **b** N 1*s*, **c** Br 3*d*, **d** I 3*d*, **e** Cs 3*d*, and **f** Pb 4*f* core-level XPS spectra for the control and target perovskite films samples prepared with 0- and 15-min ambient-air exposure. GIWAXS images of **g** control perovskite film obtained with 0-min ambient-air exposure and **h** target film produced with 15-min ambient-air exposure

Since a BABr shielding layer is introduced upon the target film, the surface composition and energy structure of the film were further studied using XPS and UPS technologies. In the C 1*s* XPS spectra (Fig. 3a), peaks at 284.8 eV correspond to C–C bonds, used as calibration, while peaks at 286.4 and 288.1 eV are associated with C–N and C = N bonds in the organic cations of the perovskite films. The shifts of the C 1*s* peaks corresponding to C–N and C = N bonds toward lower binding energies for the target films are attributed to the presence of BA^+^ cations [42, 48]. The N 1*s* XPS spectra in Fig. 3b show peaks around 400.3 and 401.8 eV for all the samples, corresponding to the C = N bonds of FA^+^ and C–N bonds of MA^+^. By comparison, the additional peaks at ~ 402.1 eV, associated with C–N bonds of BA^+^ cations [48], appear for only the target samples. These observations suggest that the BABr shielding layer remains in the final perovskite films, likely forming 2D perovskites at their surface or incorporating their bulk [42, 48]. Further analysis of the Br 3*d*, I 3*d*, Cs 3*d*, and Pb 4*f* XPS spectra (Fig. 3c–f) reveals consistent shifts in the XPS peaks of the target samples, indicating changes in their chemical environments. Notably, the shift of Pb 4*f* peaks to lower binding energies is linked to the effective coordination of BABr with Pb^2+^, which also affects the Cs 3*d* binding energy. Shifts in Br 3*d* and I 3*d* spectra may be attributed to N–H···I/Br hydrogen interactions, potentially impacting the Pb–I/Pb–Br bond lengths [49, 50]. To further clarify the existence form of BABr species, we performed GIWAXS tests upon the control perovskite film with 0-min ambient-air exposure and the target one with 15-min exposure, with a small incident angle of 0.1° [42]. As shown in Fig. 3g, h, the signals at q_xy_ = 1.01, 1.43, 1.75, and 2.20 Å⁻^1^ can be assigned to (100), (110), (111), and (200) planes of 1.68 eV-bandgap perovskite FA_0.65_MA_0.2_Cs_0.15_PbI_2.4_Br_0.6_ material, while signals at q_xy_ = 0.38 and 0.90 Å⁻^1^, corresponding to PbI_2_ impurity phases and PbI_x_–H_2_O complexes, are only detected in the control sample. All these observations are in line with the XRD results. No signals indicative of 2D perovskite were observed in either sample, suggesting that the 2D perovskite species weren’t produced in the target sample. We also delaminated the representative control and target films from the SnO_2_/ITO substrates (Fig. S9a) and performed XPS analysis to examine their compositions. As shown in Fig. S9b, the N 1*s* XPS peak corresponding to the signal of BA⁺ cations can still be detected in the target 15-min sample. These results disclose that the BABr species are more likely to incorporate into the final perovskite films. This finding contrasts with previous studies, which reported that 2D perovskites predominantly form on the surface of perovskite films, contributing to a 2D-3D heterojunction and passivating surface defects [42, 43, 48, 51]. We attribute this discrepancy to significant grain growth and coarsening during the thermal annealing of the target sample, leading to the significant diffusion of BA^+^ cations and Br⁻ anions. The BABr dopants could passivate the bulk defects of perovskite by carbonyl group (C = O) and ammonium (–NH_3_^+^) groups bonding with uncoordinated Pb^2+^, as well supported by the shifted Br 3*d*, I 3*d*, Cs 3*d*, and Pb 4*f* XPS spectra above, which could contribute to the reduced non-radiative recombination in the target films and final PSCs [41, 42, 48, 51].

### Crystallization Dynamics of Perovskite Films

**Fig. 4:**
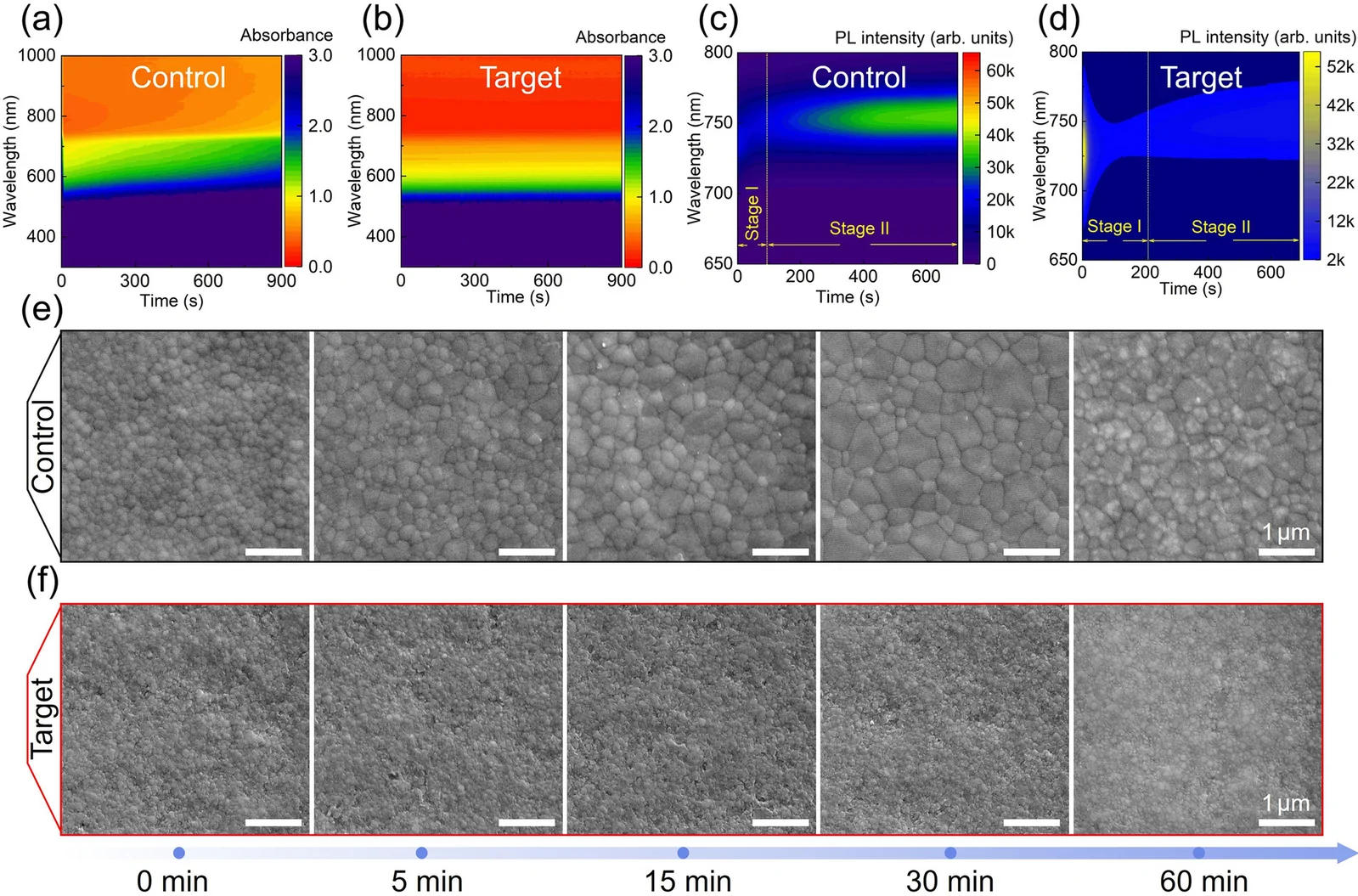
**a, b** In situ UV–vis absorption spectra and **c, d** In situ PL spectra of the control and target intermediate-phase films exposed to ambient air with RH of 60–80%. **e, f** SEM images of the intermediate-phase films with the ambient-air exposure duration of 0, 5, 15, 30, and 60 min

In situ fluorescence scattering absorption spectra and PL tests were performed to investigate the effects of slow intermolecular exchange achieved by self-buffered molecular migration strategy on the growth dynamics of the perovskite films. As shown in Fig. 4a, the absorption spectra of the control sample evolve with ambient-air standing time, showing consistent increases in absorption intensity, which indicates the spontaneous crystallization of intermediate-phase film without the BABr shielding layer. In the target sample, the influence of ambient-air exposure on absorption features is significantly weaker (Fig. 4b), suggesting that the spontaneous crystallization is largely suppressed [52]. The in situ PL results reveal crystallization dynamics for both samples. The in situ PL results could give more crystallization dynamic information of the samples. As seen from Figs. 4c, d and** S10**, with the extending of ambient-air exposure, the PL peak variations can be divided into two basic stages. In Stage I, the control and target films possess the PL peaks at 736 and 728 nm, respectively, and their intensities decrease with the exposure time, while their PL peaks shift to 754 and 749 nm in second stage (Stage II), respectively, and their intensities increase with the exposure time. The Stage I is mainly associated with the diffusion of ambient humidity molecules into the intermediate-phase film and thus the dominated nucleation process of perovskite grains [24, 36], while the Stage II is mainly related to the escape the residual solvent molecules from the film, companied by further nucleation and growth of grains [28, 39, 51], coupled with significant mass transportation. The timescales of Stage I are estimated to 96 and 216 s for control and target samples, respectively, with the target sample showing a longer nucleation time. Moreover, the weaker PL peak shifts in Stage II for the target sample suggest that nucleation remains dominant in this stage. This can be attributed to the BABr shielding layer, which not only retards the entry of ambient moisture but also hinders the escape of residual solvents from the film. Thus, one can make clear that the slow intermolecular exchange realized by self-buffered molecular migration strategy primarily functions to widen the nucleation time and humidity windows of intermediate-phase film in ambient air.

SEM images of control and target intermediate-phase samples at different ambient-air exposure durations (0, 5, 15, 30, and 60 min) in Fig. 4e, f reveal significant differences in particle size evolution. Under 0-min ambient-air exposure, the extremely larger particles, namely the fewer nucleation sites, can be identified for the control sample. Moreover, the particle sizes increase obviously with the exposure time. When it reaches to 60 min, some impurity species with bright contrast can be seen. These observations tell that the grain growth and mass transportation dominantly happen during the unannealed control sample being placed in ambient air. By contrast, the particle sizes during the whole test period are much smaller for the unannealed target sample. And even, the particle seems to tend to become smaller slightly with exposure time extending from 0 to 30 min. These results manifest that the nucleation process is more prominent in the unannealed target sample, with an increased number of nucleation sites generated by appropriately extended ambient-air exposure [53, 54].

### Improved Performance of PSCs by Self-Buffered Molecular Migration

**Fig. 5:**
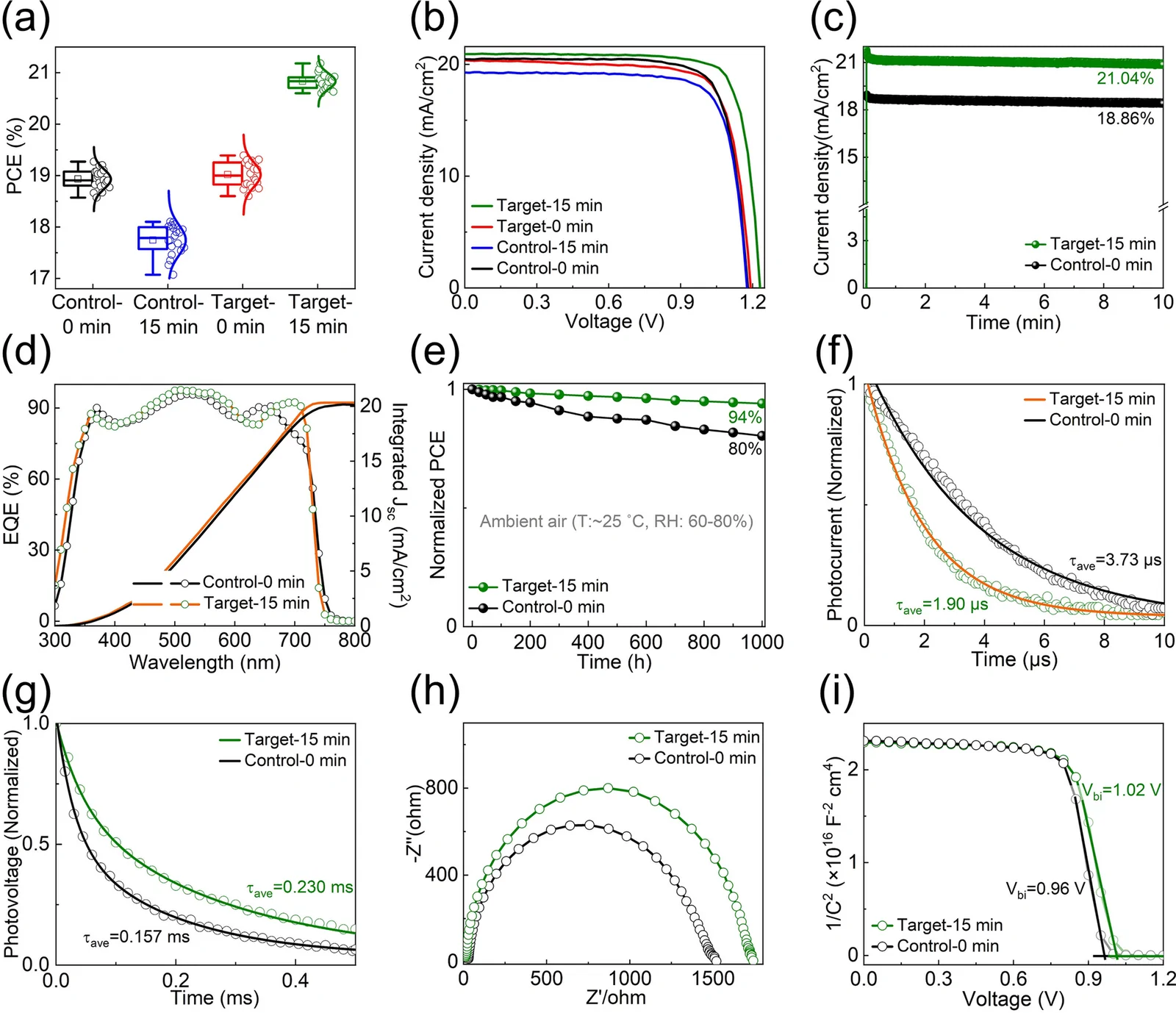
**a** Statistical RS PCEs of control and target PSCs fabricated with 0- and 15-min ambient-air exposure. **b** RS J–V curves of the champion control and target PSCs. **c** MPPT results and **d** EQE spectra of the best-performing control PSC (0-min ambient-air exposure) and target PSC (15-min ambient-air exposure). **e** Environmental stability test results measured at 60–80% RH and ~ 25 °C ambient air for the control PSC (0-min ambient-air exposure) and target PSC (15-min ambient-air exposure). **f** TPC, **g** TPV, **h** EIS, and **i** M-S analysis results for the typical control PSC (0-min ambient-air exposure) and target PSC (15-min ambient-air exposure)

To illustrate effects of slow intermediate-phase intermolecular exchange triggered by self-buffered molecular migration strategy on PSC performance, devices with an n-i-p structure (ITO/SnO_2_/perovskite/Spiro–OMeTAD/Ag) were fabricated using both control and target samples, which underwent 0 and 15 min of ambient-air exposure, respectively, under conditions of 60%–80% RH. The devices were still labeled as control and target PSCs. The corresponding photovoltaic performance parameters of 20 individual PSCs are given in Figs. 5a and** S11**. As shown in Fig. 5a, the average RS PCEs of 20 individual PSCs were determined to be (18.70 ± 0.18)% and (17.73 ± 0.29)% for the control PSCs with 0- and 15-min ambient-air exposure. In contrast to previous works [31, 46], our control PSCs exhibit relatively lower PCEs, which can be attributed to the high RH environment in which they were fabricated. When the ambient RH was reduced to 30%–40%, the control PSCs fabricated with 0- and 15-min ambient-air exposure yield enhanced average RS PCEs of (19.24 ± 0.12)% and (18.12 ± 0.15)%, respectively (Fig. S12). In comparison, the target devices showed further improvement, with average RS PCEs of (19.02 ± 0.23)% and (20.83 ± 0.14)% for the 0- and 15-min exposure samples, respectively. The latter one is higher than the devices directly annealed in glovebox with the average RS PCE of (18.90 ± 0.19)% (Fig. S13). These results indicate that with the assistance of BABr shielding layer, the beneficial effects of ambient-air, moisture-assisted thermal annealing can be achieved under high RH condition.

The J–V curves of the best-performing PSCs, measured under simulated AM 1.5G illumination, are presented in Fig. 5b, with detailed photovoltaic performance parameters listed in Table S1. All performance parameters for the target PSCs are found to be improved. Specifically, the target PSC with 15-min ambient-air exposure exhibits a *J*_sc_ of 20.96 mA cm^−2^, *V*_oc_ of 1.231 V, FF of 82.16%, and a PCE of 21.20%, positioning it among the top-performing 1.68 eV-bandgap PSCs [55, 56]. The fabrication process for the control PSCs with 0-min exposure is similar to that of previous air annealing methods. Therefore, the following investigations use the resulting PSCs as control samples, with the target PSCs (15-min ambient-air exposure) selected for comparison due to their superior PCEs. Figure 5c shows the MPPT results for the best-performing control and target PSCs, with stabilized PCEs of 18.86% and 21.04%, respectively. The EQE spectra in Fig. 5d reveal a consistent response onset wavelength of approximately 740 nm for both PSCs, further confirming the 1.68 eV wide bandgap of the control and target perovskite films. The integrated *J*_sc_ values derived from the EQE spectra are 20.02 and 20.33 mA cm^−2^ for the best-performing control and target PSCs, respectively, which align well with the values obtained from the RS J–V curves. Furthermore, the PCE evolutions for typical control and target PSCs, unencapsulated and measured in ambient air with controlled RH of 60%–80% and temperature of 25 °C, were also monitored over time. After about 1000 h of testing, the control device retained 80% of its initial PCE, whereas the target device maintained 94% (Fig. 5e), demonstrating the better operational stability of the latter. Additionally, when similar control and target devices were exposed to a temperature of 85 °C for 24 h on a hotplate inside a glovebox, they retained 89% and 95% of their initial PCEs, respectively (Fig. S14), further confirming the improved thermal stability of the target device. The better stability is primarily attributed to the enhanced resistance of the target perovskite film to humidity, thermal, and light stresses.

The carrier transport characteristics of the typical control and target PSCs are further compared to provide insight into the PCEs of the latter. The TPC results in Fig. 5f reveal a faster photocurrent decay in the target PSC (1.90 μs) compared to the control PSC (3.73 μs), indicating more efficient charge carrier extraction and transport, which likely contributes to a larger *J*_sc_ [44, 46, 47]. Additionally, when comparing the TPV decay times, the target PSC exhibits a slower decay (0.230 ms) than the control PSC (0.157 ms) in Fig. 5g. This slower decay suggests reduced non-radiative recombination of charge carriers in the target PSC, leading to higher *V*_oc_ and *J*_sc_ [44, 46, 47]. EIS and M-S analysis results in Fig. 5h, i further corroborate these findings. The larger recombination resistance (*R*_rec_) observed in the target PSC, as indicated by the semicircle in the EIS plot, is associated with weaker carrier recombination. Furthermore, the target PSC shows a higher built-in potential (*V*_bi_) of 1.02 V, compared to 0.97 V in the control PSC, which not only suggests a greater driving force for charge transport but also a broader depletion region that helps suppress carrier recombination [44, 46, 47]. Overall, these enhanced carrier dynamics in the target PSC contribute to its superior performance. These advantageous properties are largely attributed to the high-quality target perovskite films, which exhibit a pure phase, large grain size, high crystallinity, and the beneficial passivation effects of the incorporated BABr species. All these characters can make for the lower defect density and thus the better carrier dynamics in final PSC, as well verified by the space charge limited current (SCLC) analyses (Fig. S15).

### Widened Processing Windows for Ambient-Air Crystallization by Self-Buffered Molecular Migration and its Versatility

**Fig. 6:**
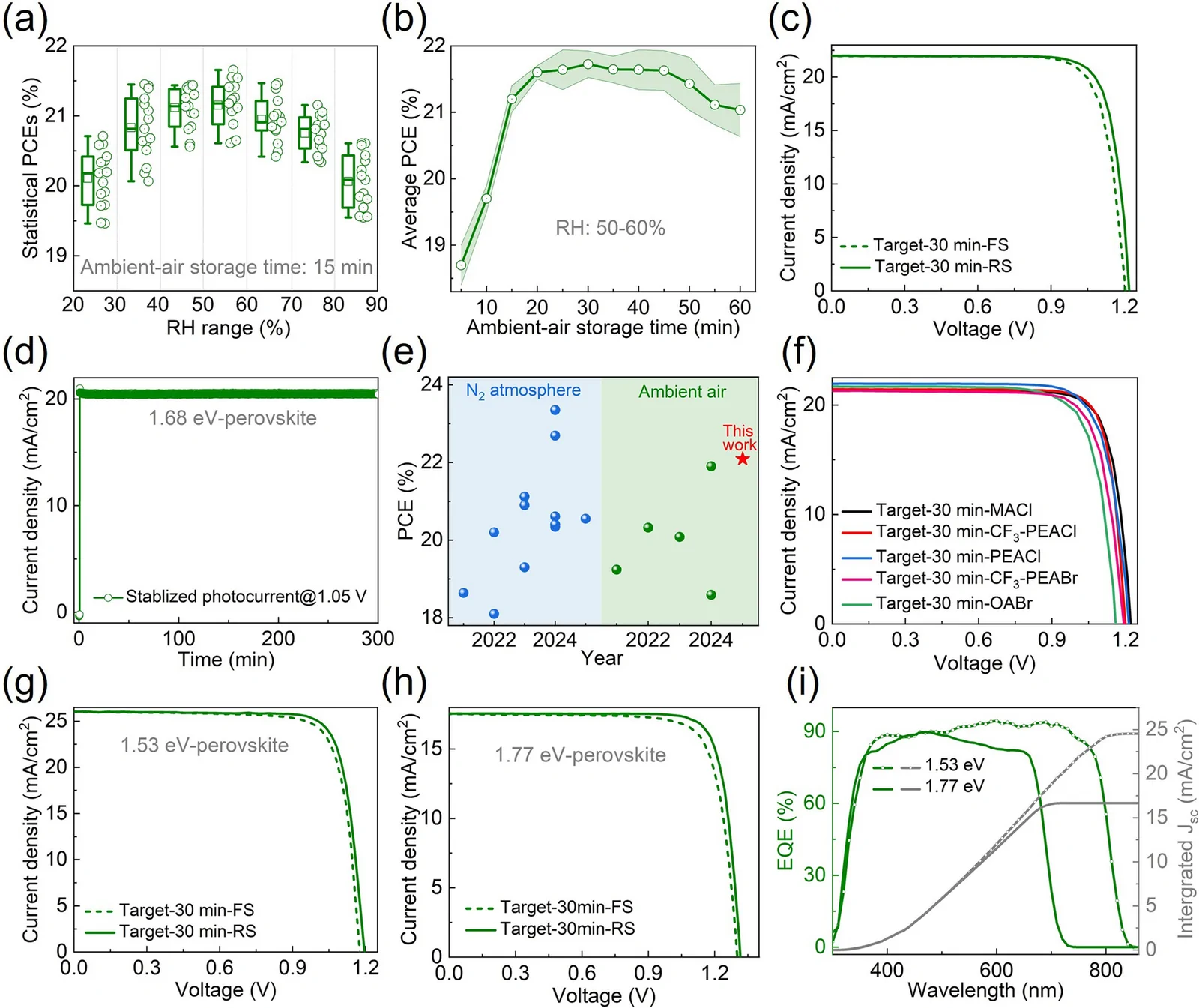
**a** Statistical RS PCEs of target PSCs fabricated with 15-min ambient-air exposure under different RH conditions. **b** Statistical RS PCEs of target PSCs fabricated under optimized RH condition but with different ambient-air exposure durations. **c** FS and RS J–V curves and **d** MPPT result for the best-performing target PSC obtained with 30-min ambient-air exposure and 50%–60% RH. **e** Summary of the historical RS PCEs of the n-i-p structured PSCs based on 1.68 eV-bandgap perovskite films prepared in N_2_-atomopshere and ambient air. **f** RS J–V curves of champion target PSCs fabricated with various shielding layers under the RH of 50%–60% and 30-min ambient-air exposure. FS and RS J–V curves for the best-performing target PSCs based on **g** 1.53 eV- and **h** 1.77 eV-bandgap perovskites fabricated under the RH of 50%–60% and 30-min ambient-air exposure. **i** EQE spectra and integrated *J*_sc_ of the champion target PSCs with 1.53 eV- and 1.77 eV-bandgap perovskites

Finally, we fabricated the target PSCs with 15-min ambient-air exposure under various RH conditions, ranging from 20%–30% to 80%–90%. As shown in Fig. 6a, PSCs with average RS PCEs exceeding 20.5% were achieved within the RH range of 30% to 80%, demonstrating an exceptionally wide RH window for fabricating high-efficiency devices. The highest average RS PCE of (21.15 ± 0.32)% was obtained at an RH of 50%–60%. Furthermore, to illustrate the broad processing time window enabled by self-buffered molecular migration strategy, the target PSCs were fabricated under the optimized RH condition of 50%–60% but with varying ambient-air exposure times ranging from 5 to 60 min. As depicted in Fig. 6b, when the exposure time was between 10 and 55 min, consistently higher PCEs are achieved for the target PSCs, indicating a wide time window for device fabrication. Notably, the optimized average RS PCE of (21.72 ± 0.24)% is achieved for PSCs stored for 30 min in ambient air Fig. 6c. The champion PSC exhibited an RS PCE of 22.09%, with a *J*_sc_ of 22.00 mA cm^−2^, *V*_oc_ of 1.220 V, and FF of 82.30% Fig. 6d. This PCE represents the highest value reported for 1.68 eV-bandgap PSCs prepared in ambient air (Table S3 and Fig. 6e), even surpassing most values obtained in N₂ atmosphere. Under forward-scan (FS) testing conditions, the champion PSC yields a PCE of 21.16%, with a *J*_sc_ of 21.94 mA cm^−2^, *V*_oc_ of 1.204 V, and FF of 80.12%, demonstrating minimal J–V hysteresis, which largely benefits from the suppressed PbI_2_ species in the corresponding perovskite films [45, 57]. Its stabilized PCE, shown in Fig. 6d, is evaluated at 21.58%, in close agreement with the PCEs derived from both RS and FS J–V curves. These results highlight that the proposed self-buffered molecular migration strategy enables a significantly wide time and RH processing window for ambient-air nucleation of perovskite films. The expanded time window enhances the producibility and scalability of PSCs [28, 39], while the reduced humidity requirements notably lower manufacturing costs [12, 14, 16].

On the other hand, it was found that, in addition to the BABr shielding layer, other materials such as MACl, CF_3_–PEACl, PEACl, CF_3_–PEABr, and OABr can also achieve the beneficial effects of the slow intermolecular exchange involved in the self-buffered molecular migration strategy [43, 58]. As shown in Fig. 6f and Table S2, wide-bandgap PSCs based on these materials, with 30-min ambient-air exposure under RH of 50%–60%, achieve the champion RS PCEs of 21.20%, 21.14%, 21.09%, 20.16%, and 20.09%, respectively. These results demonstrate the excellent versatility of the self-buffered molecular migration strategy. Furthermore, we also extend this promising strategy to the widely studied 1.53 eV-bandgap Cs_0.025_MA_0.075_FA_0.90_PbI_3_ and 1.77 eV-bandgap FA_0.8_Cs_0.2_Pb(I_0.6_Br_0.4_)_3_ perovskite films. As seen from Figs. S16 and S17, similar with the 1.68 eV-bandgap one, the grain growth and mass transportation can be significantly suppressed in the target 1.53 eV- and 1.77 eV-bandgap intermediate-phase films, revealing the similarly slow intermolecular exchange process achieved by self-buffered molecular migration strategy in them. The resulting PSCs with the 1.53 eV-bandgap films deliver exceptionally high average RS and FS PCEs of (24.40 ± 0.59)% and (23.92 ± 0.44)%, respectively (Fig. S18). The best-performing device yields the impressive RS and FS PCEs of up to 25.23% (*V*_oc_ = 1.197 V, FF = 80.86%, *J*_sc_ = 26.07 mA cm^−2^) and 24.45% (*V*_oc_ = 1.177 V, FF = 79.81%, *J*_sc_ = 26.03 mA cm^−2^), respectively (Fig. 6g), both of which surpass most ambient-air processed PSCs reported to date [39, 53, 59]. In particular, the devices based on the 1.77 eV-bandgap films exhibited average RS and FS PCEs of (18.47 ± 0.43)% and (17.68 ± 0.44)%, respectively (Fig. S19). The champion device achieves RS and FS PCEs of 19.09% (*V*_oc_ = 1.320 V, FF = 82.34%, *J*_sc_ = 17.56 mA cm^−2^) and 18.03% (V_oc_ = 1.307 V, FF = 78.68%, *J*_sc_ = 17.53 mA cm^−2^), respectively (Fig. 6h), also ranking among the highest PCEs for PSCs with 1.77 eV-bandgap perovskite films [60–63]. As shown in Fig. 6i, the photo-response onset wavelengths for the corresponding PSCs with 1.53 eV- and 1.77 eV-bandgap perovskite films are estimated to be 810 and 700 nm, respectively, in accordance with the bandgaps of Cs_0.025_MA_0.075_FA_0.90_PbI_3_ and FA_0.8_Cs_0.2_Pb(I_0.6_Br_0.4_)_3_ perovskite films. The integrated *J*_sc_ values are 24.55 and 16.68 mA cm^−2^ for the champion PSCs with 1.53 eV- and 1.77 eV-bandgap perovskite films, respectively, which are consistent with those obtained from the J–V curves. These experiments further confirm the universality of self-buffered molecular migration strategy in achieving high-efficiency PSCs with a variety of perovskite materials.

## Conclusion

The slow intermolecular exchange between perovskite intermediate-phase films and ambient humidity can be effectively achieved by self-buffered molecular migration strategy through coating a BABr shielding layer, which significantly reduces the diffusion of ambient moisture into the intermediate-phase film. This layer minimizes the sensitivity of the intermediate-phase films to ambient moisture and broadens the ambient-air nucleation time and humidity windows for perovskite crystallization, simultaneously. As a result, high-quality 1.68 eV-bandgap perovskite films with pure phase, fewer defects, larger-sized grains, and better stability are obtained, even under extended ambient-air exposure periods and high RH condition of 60%–80%. Utilizing these high-quality perovskite films, wide-bandgap n-i-p structured PSCs achieve a significantly enhanced average RS PCE of (21.72 ± 0.24)%, along with superior humidity stability. In particular, the optimized 1.68 eV-bandgap PSC, obtained under ambient-air exposure time of 30 min and RH of 50–60%, yields a record-high PCE of 22.09%, the highest reported for ambient-air processed 1.68 eV-bandgap devices. Meanwhile, the versatility of the proposed self-buffered molecular migration strategy is demonstrated by use of various shielding materials, including MACl, CF_3_–PEACl, PEACl, CF_3_–PEABr, and OABr. Its universality is further confirmed by applying the strategy to 1.53 eV- and 1.77 eV-bandgap perovskite materials, resulting in the impressive RS PCEs of 25.23% and 19.09%, respectively, for n-i-p structured PSCs.

## Supplementary Information

Below is the link to the electronic supplementary material.

Supplementary file1 (DOCX 3063 KB)
