# Supplementary material for: Moisture-Resistant Scalable Ambient-Air Crystallization of Perovskite Films via Self-Buffered Molecular Migration Strategy
Source: Nanomicro Lett. 2025 Sep 1;18:53. doi: 10.1007/s40820-025-01851-9 (PMC12401818; doi:10.1007/s40820-025-01851-9)
Supplement: Supplementary file 1 — Supplementary file1 (DOCX 3063 KB) [file 40820_2025_1851_MOESM1_ESM.docx]

Supporting Information for

**Moisture-Resistant Scalable** **Ambient-Air Crystallization of Perovskite Films via** **Self-Buffered Molecular Migration Strategy**

Mei Yang^1^, Weidong Zhu^1*^, Laijun Liang^1^, Wenming Chai^1^, Xiaomeng Wu^2*^, Zeyang Ren^1^, Long Zhou^3^, Dazheng Chen^1^, He Xi^3^, Chunfu Zhang ^1*^, Jincheng Zhang^1^, Yue Hao^1^

^1^State Key Laboratory of Wide-Bandgap Semiconductor Devices and Integrated Technology, Xidian University, Xi'an 710071, P. R. China

^2^School of Electronic Engineering, Xi’an Shiyou University, Xi’an 710065, P. R. China

^3^School of Advanced Materials and Nanotechnology, Xidian University, Xi’an 710126, P. R. China

*Corresponding authors. E-mail: wdzhu@xidian.edu.cn (Weidong Zhu); xmwudz@xsyu.edu.cn (Xiaomeng Wu); cfzhang@xidian.edu.cn (Chunfu Zhang)

**Supplementary Figures and Tables**


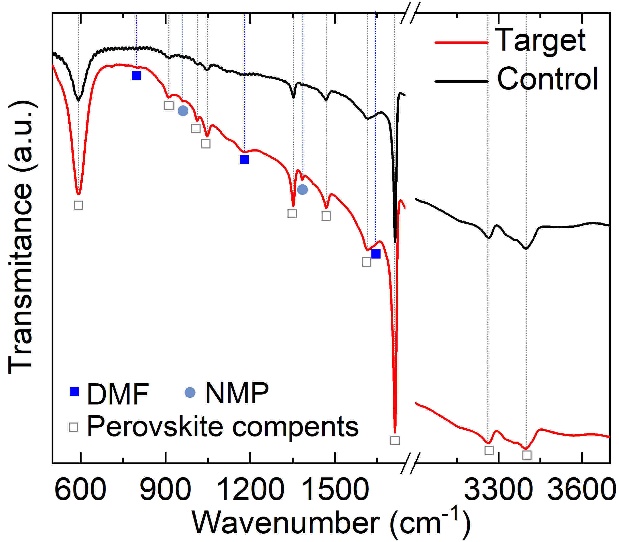


**Fig. S1** FTIR spectra of control and target intermediate-phase films after being exposed to ambient air for about 120 min


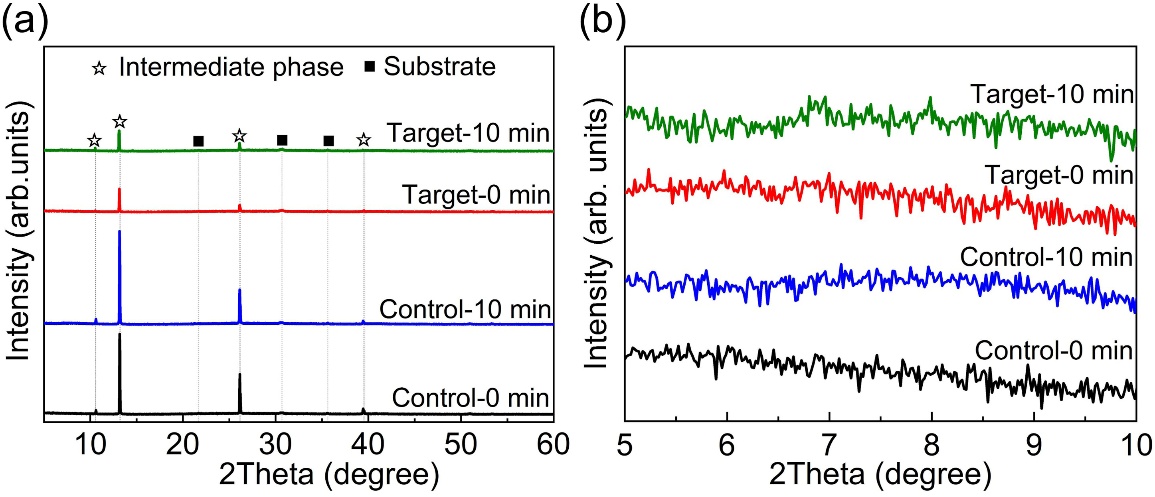


**Fig. S2 a** XRD patterns and **b** the magnified results in the range 2θ = 5°–10° for the control and target intermediate-phase films just exposure to ambient air (Conrol-0 min and Control-10 min) and exposed for 10 min (Target-0 min and Target-10 min)

**
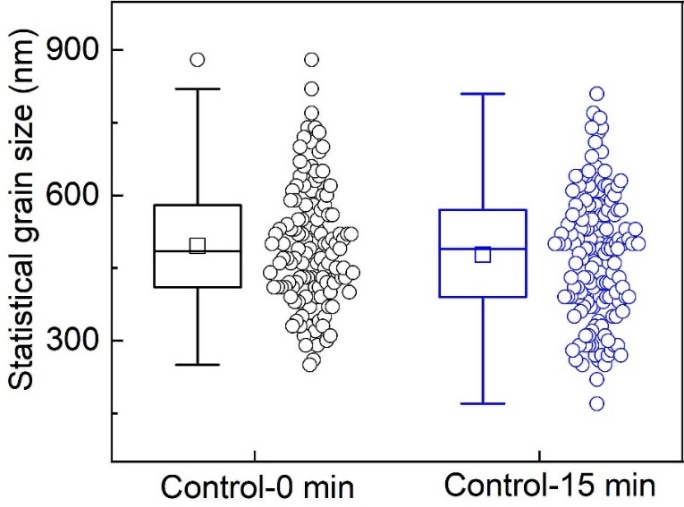
**

**Fig. S3** Statistical grain sizes of control and target perovskite films obtained with 0- and 15-min ambient-air exposure


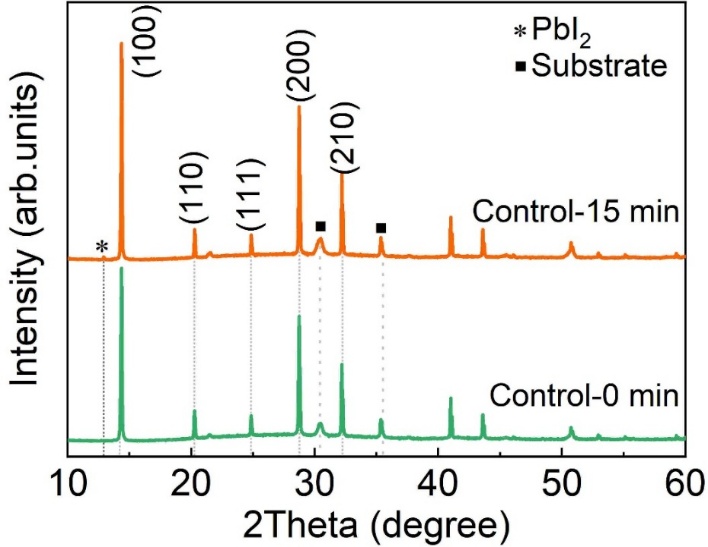


**Fig. S4** XRD patterns of the control perovskite films obtained with 0- and 15-min ambient-air loading and prepared in ambient air with RH of below 20%


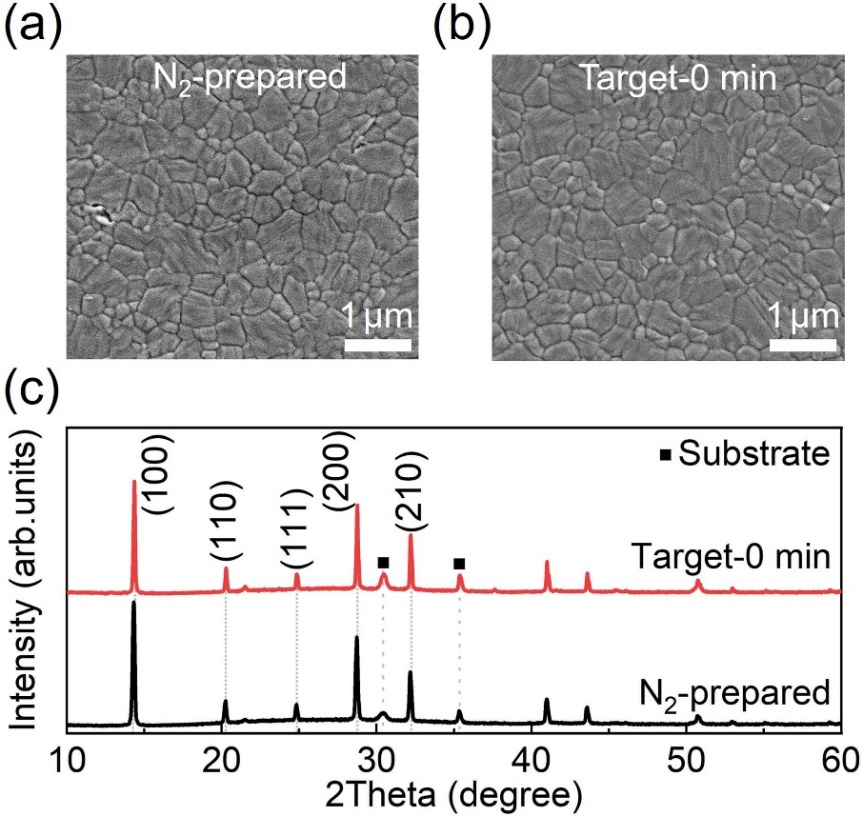


**Fig. S5** SEM images of **a** the perovskite film produced in glovebox (labeled as N_2_-preapred) and **b** the target perovskite film prepared with 0-min ambient-air exposure. **c** The corresponding XRD patterns


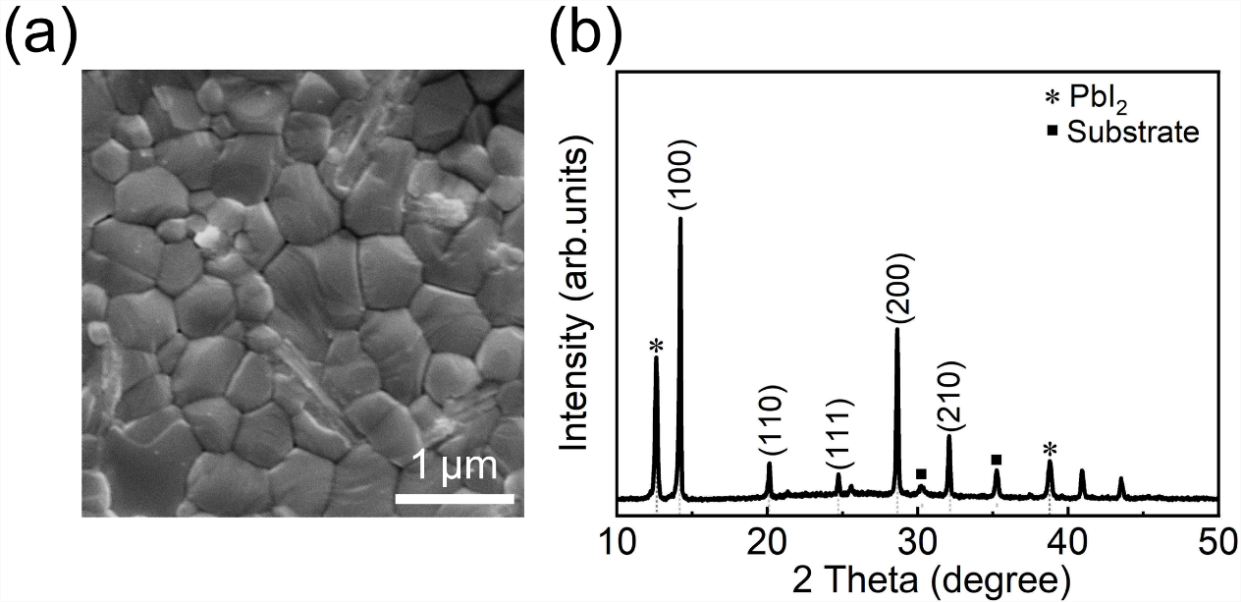


**Fig. S6 a** SEM image and **b** XRD pattern of perovskite film prepared by use of pure IPA solvent and with 0 min ambient-air exposure before annealing


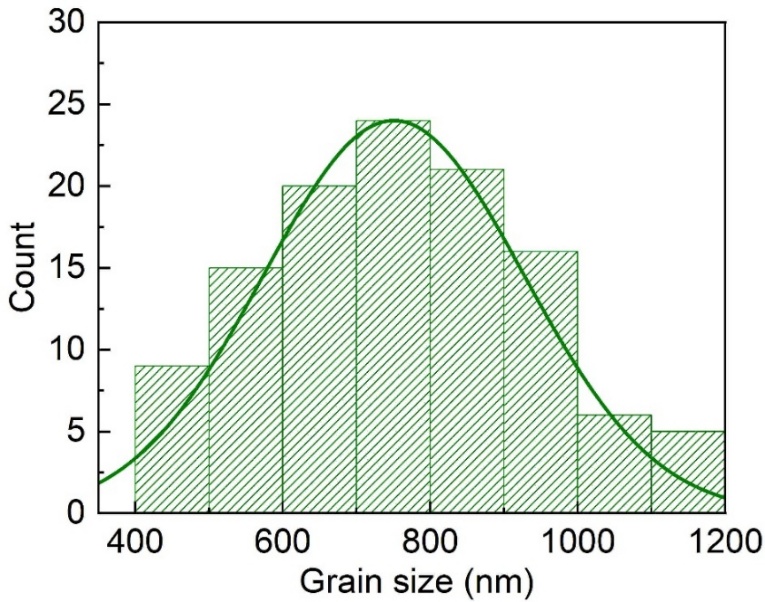


**Fig. S7** Statistical grain sizes for the target sample obtained with 15-min ambient-air exposure


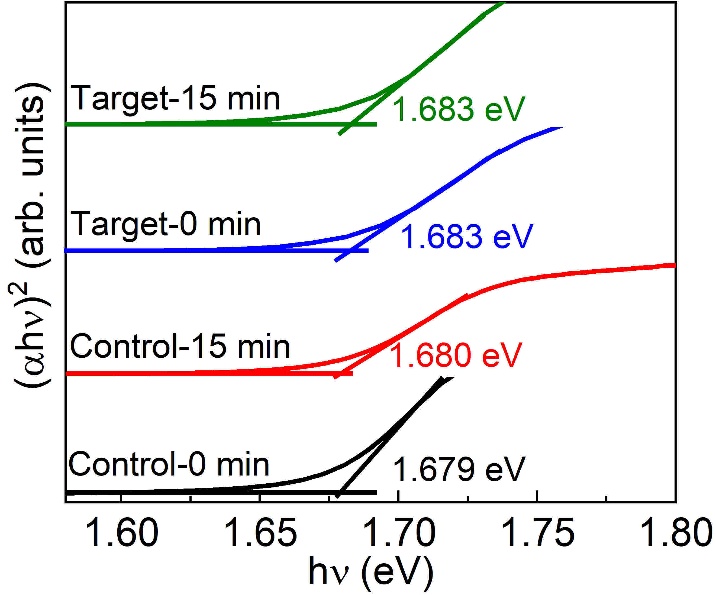


**Fig. S8** Tauc plots derived from the absorption spectra of the control and target perovskite films obtained with 0- and 15-min ambient exposure


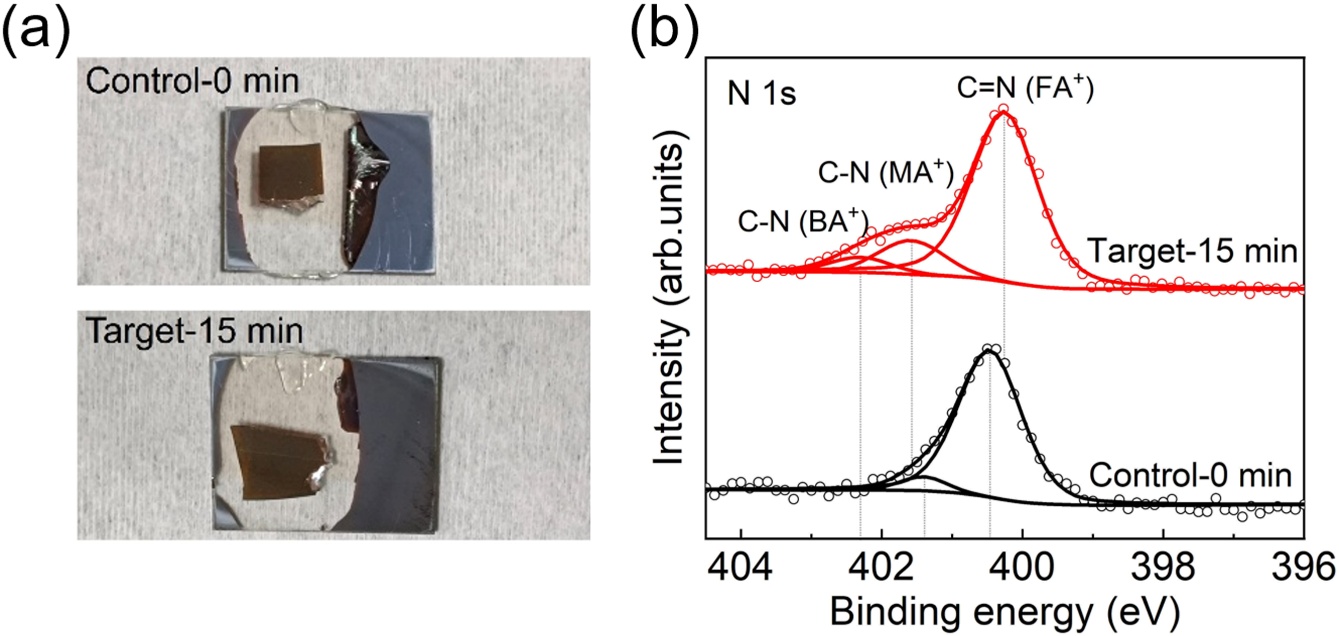


**Fig. S9** **a** Photographs and **b** core-level N 1s spectra of the delaminated control-0 min and target-15min films


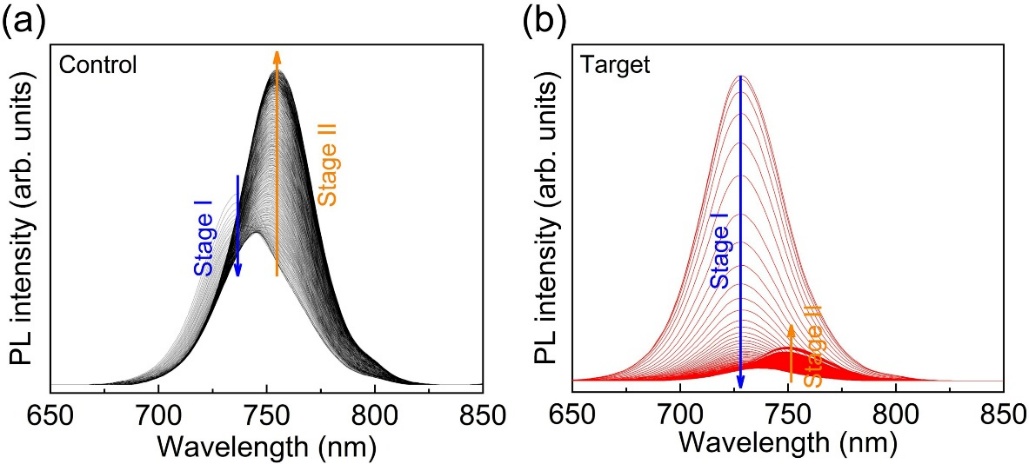


**Fig. S10** Re-plotted in-situ PL spectra for **a** control and **b** target intermediate-phase films exposed to ambient air with RH of 60-80%


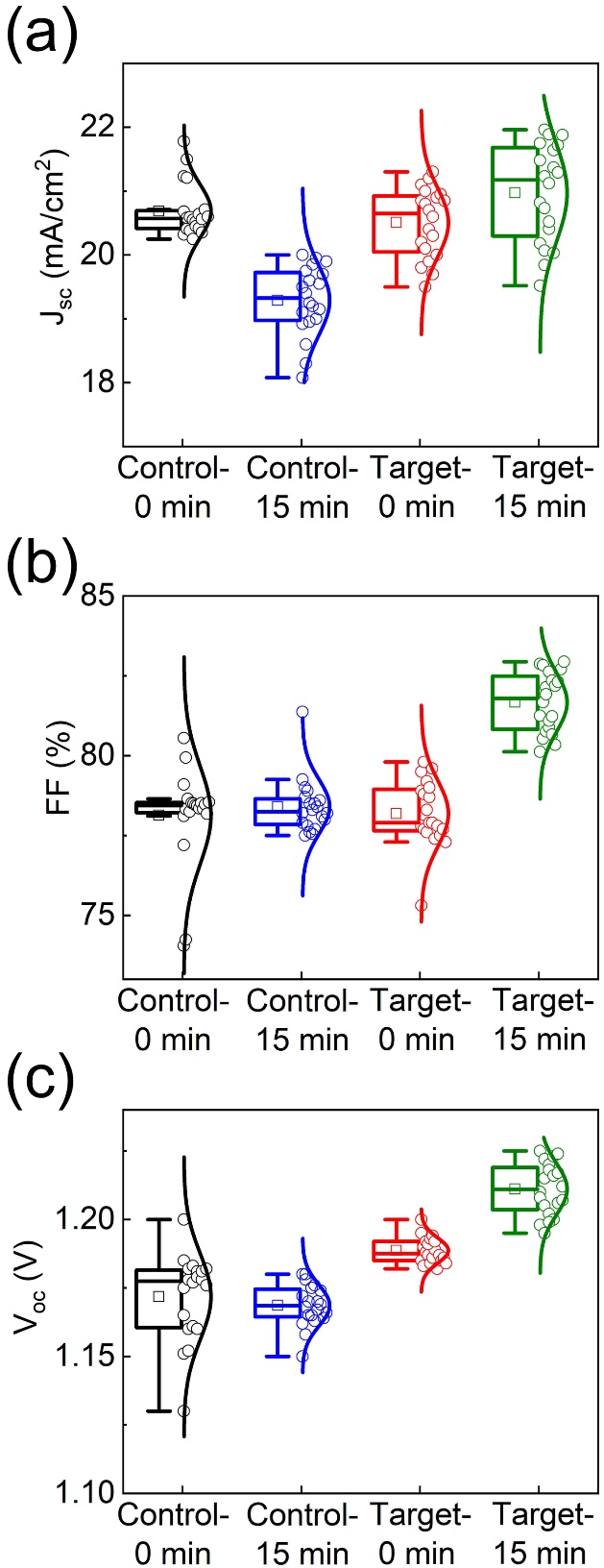


**Fig. S11** Statistical **a** J_sc_s, **b** FFs, and **c** V_oc_s for 20 individual control and target PSCs fabricated with 0- and 15-min ambient-air exposure


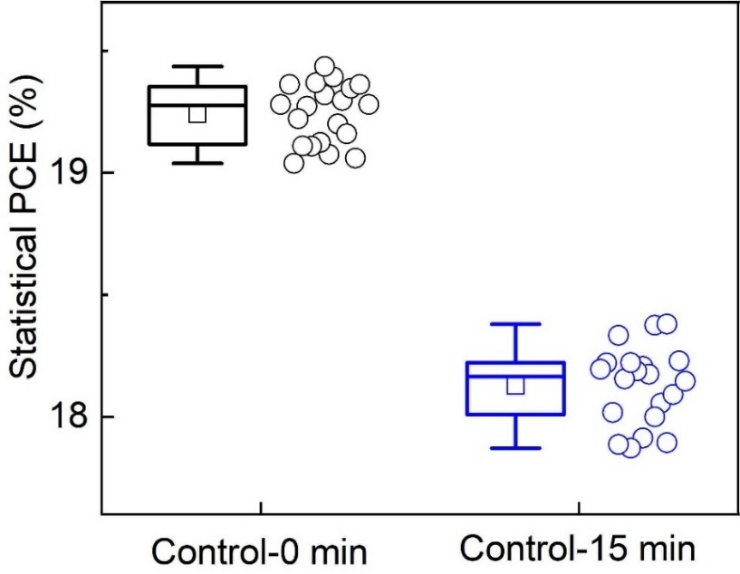


**Fig. S12** Statistical RS PCEs for the control PSCs fabricated with 0- and 15-min ambient-air exposure. During the device fabrication process, the RH of ambient air was controlled to 30~40%


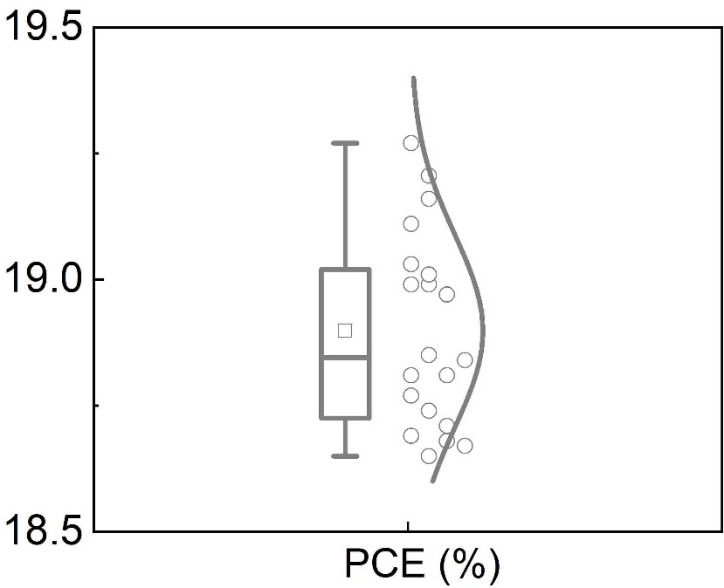


**Fig. S13** Statistical RS PCEs for the PSCs fabricated in glovebox and with coating of BABr layer

**
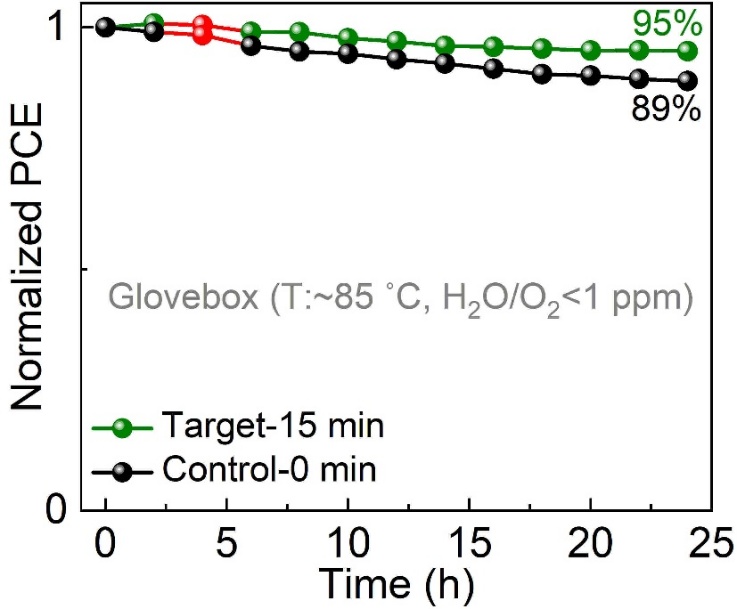
**

**Fig. S14** Thermal stability test results measured at ~85 ℃ and glovebox (H_2_O/O_2_ < 1ppm) for the control PSC (0-min ambient-air exposure) and target PSC (15-min ambient-air exposure)


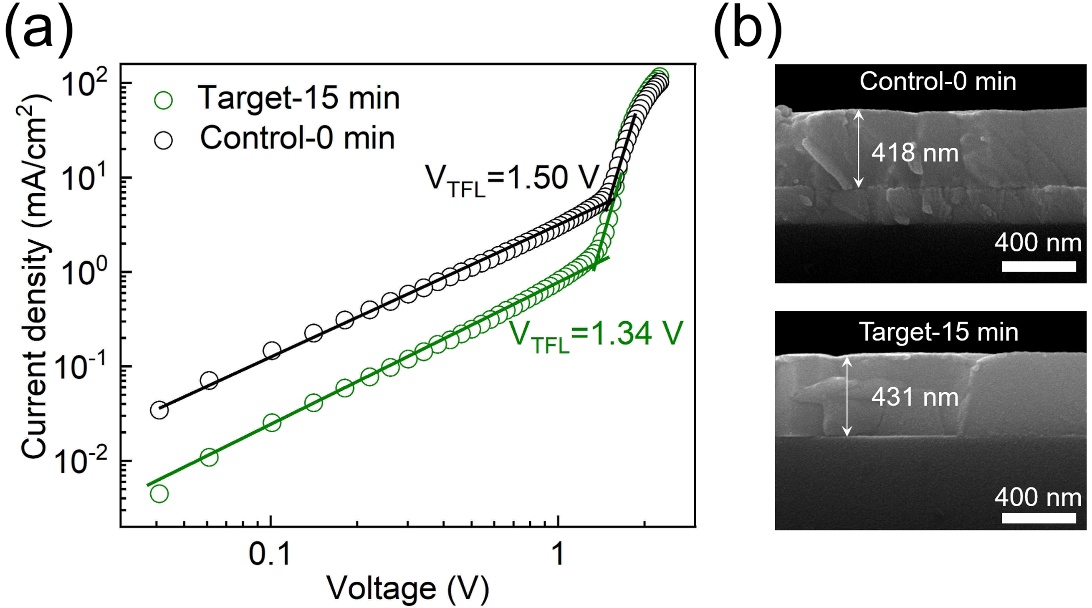


**Fig. S15 a** SCLC analysis of hole-transport-only devices (ITO/NiO_x_/perovskite/Spiro-OMeTAD/Ag) based on the control film (0-min ambient-air exposure) and target film (15-min ambient-air exposure). **b** Cross-sectional SEM images of control film (0-min ambient-air exposure) and target film (15-min ambient-air exposure)

From **Fig. S15**(a), the trap-filled limit voltages (V_TFL_s) were determined to 1.50 and 1.34 V for control film (0-min ambient-air exposure) and target film (15-min ambient-air exposure), respectively. The trap density (N_t_) can be correlated with the V_TFL_ by following N_t_=2V_TFL_ε_r_ε_0_/eL^2^, for which L is the thickness of perovskite film that can be determined from the cross-sectional SEM images in **Fig. S15**(b), ε_0_ stands for the vacuum permittivity (8.8542×10^-14^ F/m), ε_r_ represents the relative dielectric constant of perovskite film (32), and e is the elementary charge (1.602×10^-19^ C). In this manner, the trap densities were determined to 3.04×10^16^ and 2.55×10^16^ cm^-3^ for the control film (0-min ambient-air exposure) and target film (15-min ambient-air exposure), respectively.


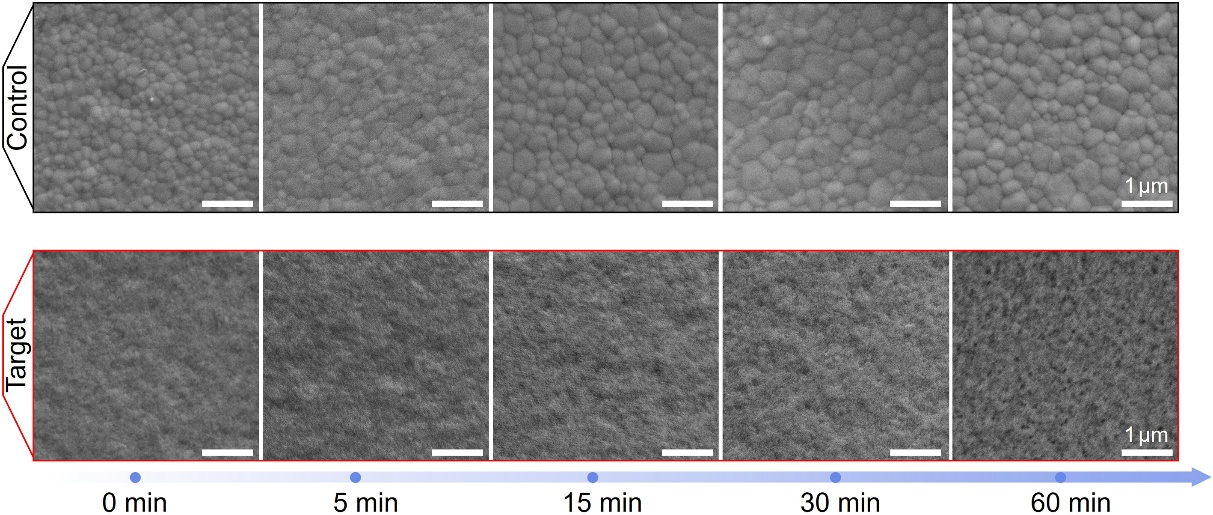


**Fig. S16** SEM images of the 1.53 eV-bandgap intermediate-phase films with the ambient-air exposure duration of 0, 5, 15, 30, and 60 min


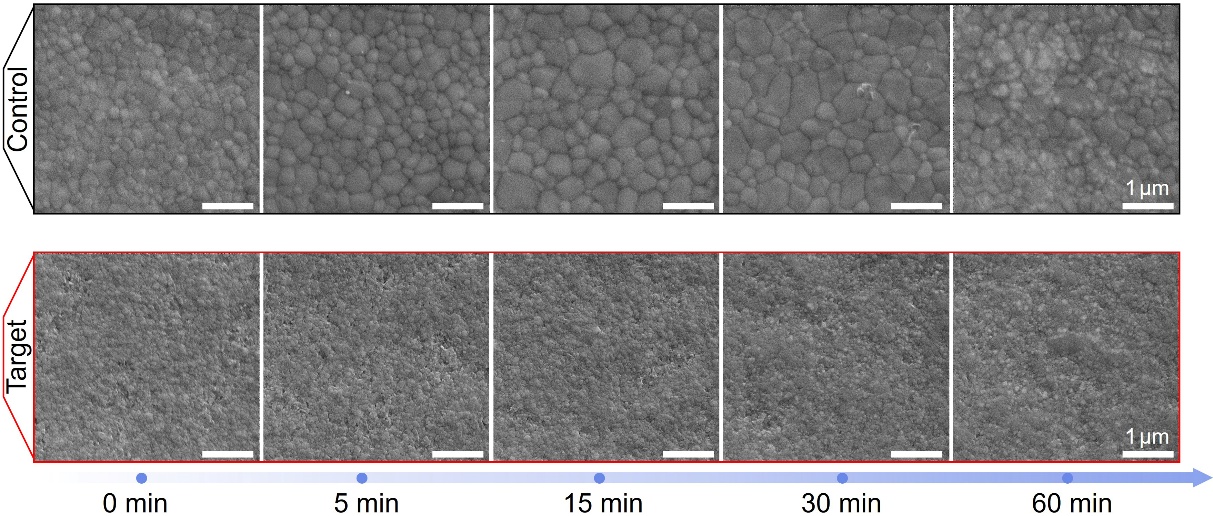


**Fig. S17** SEM images of the 1.77 eV-bandgap intermediate-phase films with the ambient-air exposure duration of 0, 5, 15, 30, and 60 min


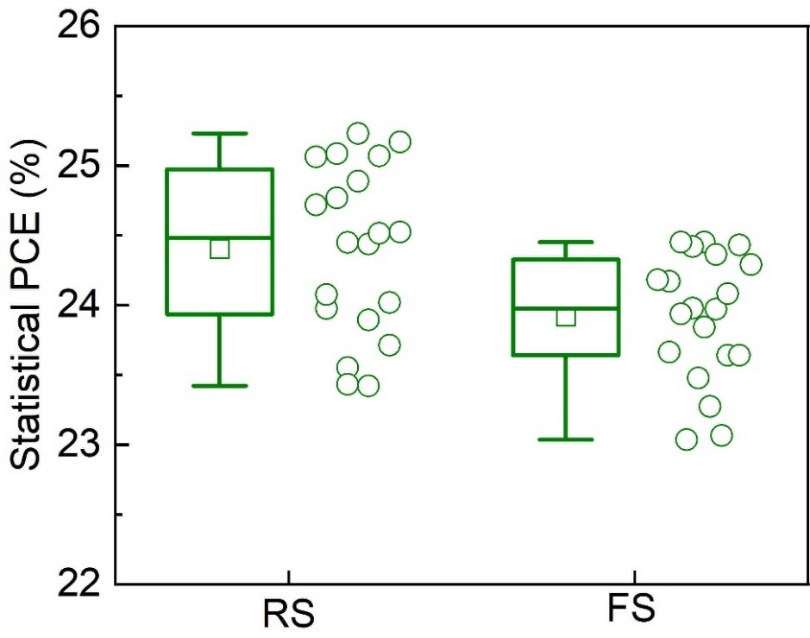


**Fig. S18** Statistical RS and FS PCEs for the target PSCs based on 1.53 eV-bandgap perovskite films


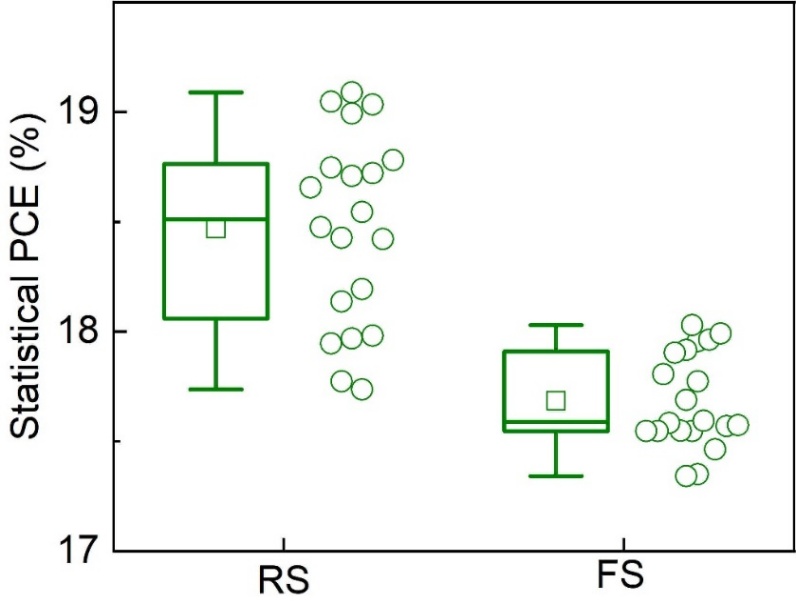


**Fig. S19** Statistical RS and FS PCEs for the target PSCs based on 1.77 eV-bandgap perovskite films

**Table S1** Photovoltaic performance parameters recorded under RS for best-performing control and target PSCs fabricated with 0- and 15-min ambient-air exposure

| Samples | J_sc_ (mA/cm^2^) | Voltage (V) | FF (%) | PCE (%) |
| --- | --- | --- | --- | --- |
| Control-0 min | 20.54 | 1.180 | 78.26 | 18.97 |
| Control-15 min | 19.31 | 1.176 | 79.09 | 17.96 |
| Target-0 min | 20.37 | 1.191 | 78.58 | 19.06 |
| Target-15 min | 20.96 | 1.231 | 82.16 | 21.20 |

**Table S2** Photovoltaic performance parameters recorded under RS for the best-performing PSCs assembled with different shielding layers and 30-min ambient-air exposure

| Samples | V_oc_ (V) | J_sc_(mA/cm^2^) | FF (%) | PCE (%) |
| --- | --- | --- | --- | --- |
| Target-30 min MACl | 1.222 | 21.43 | 80.97 | 21.20 |
| Target-30 min CF_3_PEACl | 1.200 | 21.47 | 82.06 | 21.14 |
| Target-30 min PEACl | 1.214 | 21.93 | 79.24 | 21.09 |
| Target-30 min CF_3_PEABr | 1.194 | 21.32 | 79.21 | 20.16 |
| Target-30 min OABr | 1.160 | 21.69 | 79.85 | 20.09 |

**Table S3** Summary of the historical RS PCEs, J_sc_s, V_oc_s, and FFs of the n-i-p structured PSCs based on 1.68 eV-bandgap perovskite films prepared in N_2_-atomopshere and ambient air

| Preparation atmosphere | V_oc_ (V) | J_sc_ (mA/cm^2^) | FF (%) | PCE (%) | Publication  year | Refs |
| --- | --- | --- | --- | --- | --- | --- |
| Ambient air | 1.220 | 22.00 | 82.3 | 22.09 | 2025 | This work |
|  | 1.2 | 22.72 | 80.3 | 21.9 | 2024 | [S1] |
|  | 1.139 | 20.73 | 78.7 | 18.59 | 2024 | [S2] |
|  | 1.255 | 20.31 | 79.0 | 20.08 | 2023 | [S3] |
|  | 1.2 | 20.68 | 81.87 | 20.32 | 2022 | [S4] |
|  | 1.22 | 20.38 | 77.22 | 19.24 | 2021 | [S5] |
| N_2_ atmosphere | 1.22 | 21.27 | 79.2 | 20.55 | 2025 | [S6] |
|  | 1.282 | 22.43 | 81.2 | 23.35 | 2024 | [S7] |
|  | 1.212 | 22.52 | 83.13 | 22.69 | 2024 | [S8] |
|  | 1.214 | 20.49 | 82.49 | 20.34 | 2024 | [S9] |
|  | 1.249 | 20.31 | 81.25 | 20.61 | 2024 | [S10] |
|  | 1.21 | 21.8 | 77.3 | 20.4 | 2024 | [S11] |
|  | 1.25 | 21.85 | 77.66 | 21.12 | 2023 | [S12] |
|  | 1.17 | 21.81 | 81.90 | 20.90 | 2023 | [S13] |
|  | 1.22 | 20.6 | 76.9 | 19.3 | 2023 | [S14] |
|  | 1.256 | 20.43 | 78.71 | 20.2 | 2022 | [S15] |
|  | 1.233 | 20.5 | 71.6 | 18.1 | 2022 | [S16] |
|  | 1.195 | 19.95 | 78.2 | 18.64 | 2021 | [S17] |

**Supplementary References**

1. T. Dong, L. Tan, Z. Li, J. Li, H. Li et al., Enhanced efficiency and intrinsic stability of wide-bandgap perovskite solar cells through dimethylamine-based cation engineering. Chem. **31**(4), e202403527 (2025). <https://doi.org/10.1002/chem.202403527>
2. T. Yang, D. Huang, Z. Wang, F. Li, J. Gong et al., Manipulating wide-band-gap perovskite compositions *via* Br sources for highly efficient perovskite/silicon tandem solar cells. ACS Appl. Energy Mater. **7**(14), 5755–5764 (2024). <https://doi.org/10.1021/acsaem.4c00822>
3. X. Zhang, X. Li, L. Tao, Z. Zhang, H. Ling et al., Precise control of crystallization and phase-transition with green anti-solvent in wide-bandgap perovskite solar cells with open-circuit voltage exceeding 1.25 V. Small **19**(22), 2208289 (2023). <https://doi.org/10.1002/smll.202208289>
4. X. Sun, Z. Shao, Z. Li, D. Liu, C. Gao et al., Highly efficient CsPbI3/Cs_1-_*_x_*DMAxPbI3 bulk heterojunction perovskite solar cell. Joule **6**(4), 850–860 (2022). <https://doi.org/10.1016/j.joule.2022.02.004>
5. B. Chen, P. Wang, R. Li, N. Ren, Y. Chen et al., Composite electron transport layer for efficient N-I-P type monolithic perovskite/silicon tandem solar cells with high open-circuit voltage. J. Energy Chem. **63**, 461–467 (2021). <https://doi.org/10.1016/j.jechem.2021.07.018>
6. X. Chen, Z. Yuan, S. Fan, X. Feng, X. Sun et al., Enhanced light transmittance of electron transport layer through bilayer SnO_2_ for high-performance semitransparent perovskite solar cells. ChemSusChem **18**(11), e202402582 (2025). <https://doi.org/10.1002/cssc.202402582>
7. Q. Ye, W. Hu, J. Zhu, Z. Cai, H. Zhang et al., Suppressing charge recombination in a methylammonium-free wide-bandgap perovskite film for high-performance and stable perovskite solar cells. Energy Environ. Sci. **17**(16), 5866–5875 (2024). <https://doi.org/10.1039/D4EE00666F>
8. D. Ding, Y. Yao, P. Hang, C. Kan, X. Lv et al., Visualizing the structure-property nexus of wide-bandgap perovskite solar cells under thermal stress. Adv. Sci. **11**(29), 2401955 (2024). <https://doi.org/10.1002/advs.202401955>
9. J. Shen, X. Ge, Q. Ge, N. Li, Y. Wang et al., Improvement of photovoltaic performance of perovskite solar cells by synergistic modulation of SnO_2_ and perovskite *via* interfacial modification. ACS Appl. Mater. Interfaces **16**(19), 24748–24759 (2024). <https://doi.org/10.1021/acsami.4c03595>
10. H. Yang, K. Wu, H. Guo, J. Wei, J. Guo et al., Crystallinity control and strain release in wide-bandgap perovskite film *via* seed-induced growth for efficient photovoltaics. ACS Appl. Mater. Interfaces **16**(32), 42566–42576 (2024). <https://doi.org/10.1021/acsami.4c08445>
11. S. Zhang, J. Wang, N. Kalasariya, P. Dally, C. Deger et al., Mitigating buried-interface energy losses through multifunctional ligands in n–i–p perovskite/silicon tandem solar cells. ACS Energy Lett. **9**(9), 4633–4644 (2024). <https://doi.org/10.1021/acsenergylett.4c01841>
12. Z. Li, X. Li, X. Feng, X. Chen, J. Chen et al., Strain control of mixed-halide wide-bandgap perovskites for highly efficient and stable solar cells. Sol. RRL **7**(22), 2300615 (2023). <https://doi.org/10.1002/solr.202300615>
13. X. Niu, N. Li, Z. Cui, L. Li, F. Pei et al., Anion confinement for homogeneous mixed halide perovskite film growth by electrospray. Adv. Mater. **35**(45), 2305822 (2023). <https://doi.org/10.1002/adma.202305822>
14. J. Zhao, A.S.R. Chesman, J. Yan, L.J. Sutherland, J. Jasieniak et al., Precursor engineering of lead acetate-based precursors for high-open-circuit voltage wide-bandgap perovskite solar cells. ACS Appl. Mater. Interfaces **15**(15), 18800–18807 (2023). <https://doi.org/10.1021/acsami.2c22179>
15. J. Tao, Z. Yu, X. Liu, J. Xue, J. Shen et al., A facile strategy to adjust SnO_2_/perovskite interfacial properties for high-efficiency perovskite solar cells. J. Mater. Chem. C **10**(21), 8414–8421 (2022). <https://doi.org/10.1039/D2TC00818A>
16. Z. Su, D. Xu, Q. Ma, K. Gao, C. Zhang et al., Atomic layer deposited ZnO–SnO_2_ electron transport bilayer for wide-bandgap perovskite solar cells. Sol. RRL **7**(3), 2201026 (2023). <https://doi.org/10.1002/solr.202201026>
17. X. Cui, P. Wang, B. Shi, Y. Zhao, X. Zhang, Insights into the effect of bromine-based organic salts on the efficiency and stability of wide bandgap perovskite. Nano Sel. **2**(3), 615–623 (2021). <https://doi.org/10.1002/nano.202000183>
